# CO_2_ Electrolysis via Surface-Engineering Electrografted Pyridines on Silver Catalysts

**DOI:** 10.1021/acscatal.2c01654

**Published:** 2022-06-17

**Authors:** Maryam Abdinejad, Erdem Irtem, Amirhossein Farzi, Mark Sassenburg, Siddhartha Subramanian, Hugo-Pieter Iglesias van Montfort, Davide Ripepi, Mengran Li, Joost Middelkoop, Ali Seifitokaldani, Thomas Burdyny

**Affiliations:** † Department of Chemical Engineering, Delft University of Technology, Van der Maasweg 9, Delft 2629 HZ, The Netherlands; ‡ Department of Chemical Engineering, McGill University, Montreal H3A 0C5, Canada

**Keywords:** carbon dioxide reduction, heterogeneous electrocatalysts, pyridine catalysts, silver electrocatalyst, electrografting

## Abstract

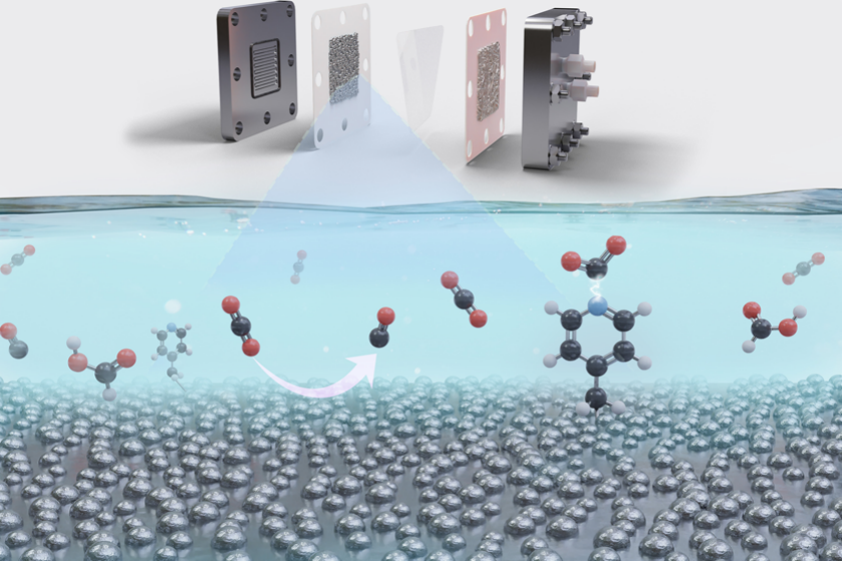

The electrochemical reduction of carbon dioxide (CO_2_) to value-added materials has received considerable attention. Both bulk transition-metal catalysts and molecular catalysts affixed to conductive noncatalytic solid supports represent a promising approach toward the electroreduction of CO_2_. Here, we report a combined silver (Ag) and pyridine catalyst through a one-pot and irreversible electrografting process, which demonstrates the enhanced CO_2_ conversion versus individual counterparts. We find that by tailoring the pyridine carbon chain length, a 200 mV shift in the onset potential is obtainable compared to the bare silver electrode. A 10-fold activity enhancement at −0.7 V vs reversible hydrogen electrode (RHE) is then observed with demonstratable higher partial current densities for CO, indicating that a cocatalytic effect is attainable through the integration of the two different catalytic structures. We extended the performance to a flow cell operating at 150 mA/cm^2^, demonstrating the approach’s potential for substantial adaptation with various transition metals as supports and electrografted molecular cocatalysts.

## Introduction

Carbon dioxide (CO_2_) is a primary contributor to global climate changes.^1^ Capture and electrochemical CO_2_ reduction reaction (CO_2_RR) to value-added feedstocks and chemicals offer a promising approach to sustainable energy storage that leverages renewable sources.^2,3^ Despite its abundancy, the CO_2_ molecule is thermodynamically stable, making its electroconversion challenging in terms of (i) competition with the hydrogen evolution reaction (HER) in an aqueous environment, (ii) low stability, and (iii) high overpotentials.^3^

To subvert activity and selectivity challenges for CO_2_ reduction, researchers have developed numerous classes of catalysts. Three classes frequently used and modified are bulk transition metals,^4^ nanoparticles,^5−8^ and molecular catalysts.^9,10^ Both seek to activate the linear CO_2_ molecule toward the desired product at enhanced reaction rates while simultaneously limiting the electrochemical activity of the competing HER. The commonly utilized transition-metal catalysts are silver and copper, with common catalytic modifications to enhance the performance occurring as a result of varying morphologies and surface areas.^11−15^ Alternatively, molecular catalysts range broadly from single-metal sites such as porphyrin and phthalocyanines^16−20^ to metal-free catalysts (e.g., pyridine),^21−23^ with modifications accessible by varying chain lengths, metal sites, and supporting ring structures. For these systems, the interactions between aqueous CO_2_ and the molecular catalyst’s ligands can act as a capturing site for CO_2_, while the designed center sites can provide the conversion step. Through characterizations and modifications, both catalytic approaches are separately able to be near-unity selective toward CO at elevated current densities, with improved efficiency and stability, which represent the key performance targets for CO_2_RR.

While both bulk transition-metal surfaces and molecular catalysts individually represent viable options for CO_2_ conversion, many of the modifications available to further increase the activity and decrease activation potentials have been well explored, providing diminishing returns on performance metrics. Alternatively, affixing a CO_2_RR active molecular catalyst onto a CO_2_RR active catalytic support approach may provide performance enhancements beyond the individual catalysts themselves by allowing for dual functionalities that overcome individual limitations. Flexibility in further modifying the collective system is then also provided. Emerging approaches to hybrid systems then have the potential to combine the two best catalyst traits of the individual systems: localizing the CO_2_ capture ability of molecular catalysts near an electrode’s surface followed by utilizing the large active area and conversion of a bulk transition metal.^12−14^

Several groups have suggested using pyridine either as a catalyst or as a promotor for the electroreduction of CO_2_;^25−32^^25−32^ however, some of these reports questioned the catalytic activity of pyridinium ions for CO_2_RR.^27,30,33,34^ For instance, on platinum^27^ or gold^35^ electrodes, the pyridinium ion was reported to act as a HER promoter as no products for the CO_2_RR were detected, whereas other studies have shown that N-substituted pyridiniums can aid the catalytic activity of Cu toward the production of C_2+_ hydrocarbons,^36,37^ or improve the catalytic selectivity of Au electrodes by facilitating proton transfer to the key intermediate, HCOO*.^38^

Here, we sought to investigate the potential for CO_2_-reactive molecular catalysts to be affixed to a CO_2_-reactive support, rather than as an aqueous species. Such a demonstration would open a new parameter space of combined catalysts to explore. A challenging aspect of a combined approach is affixing a molecular catalyst close enough to a transition-metal catalyst so that they do not function independently. Without this, no advantage can reasonably be expected over the separate cases.

From these inclinations, we hypothesized that the integration of an *N*-based molecular catalyst traditionally used in homogeneous environments would pair well with a heterogeneous silver electrocatalyst for a number of reasons. First, *N*-based molecular electrocatalysts such as arylpyridiniums are able to cocatalyze CO_2_RR^39,40^ but have demonstrated poor electroreduction of CO_2_ in homogeneous environments, albeit at low rates and selectivities.^26,27,41−43^ Second, due to the direct interaction between the −*N* group and the electrode surface, both the electrode surface and the pyridine catalytic active site (−*N* group) may be blocked in homogeneous media,^44,45^ hence decreasing the overall catalytic activity of the system for CO_2_RR. Third, pyridine can be modified with various chain lengths, which, if affixed to a heterogeneous support, allows for the distance between the electrode surface and the CO_2_ capture site of the pyridine ring to be controlled and tuned. To ensure a bond between the ligand of the pyridine molecular catalyst and the silver support that can withstand a reducing potential, the novel field of electrografting shows promise.^46,47^

**Figure 1:**
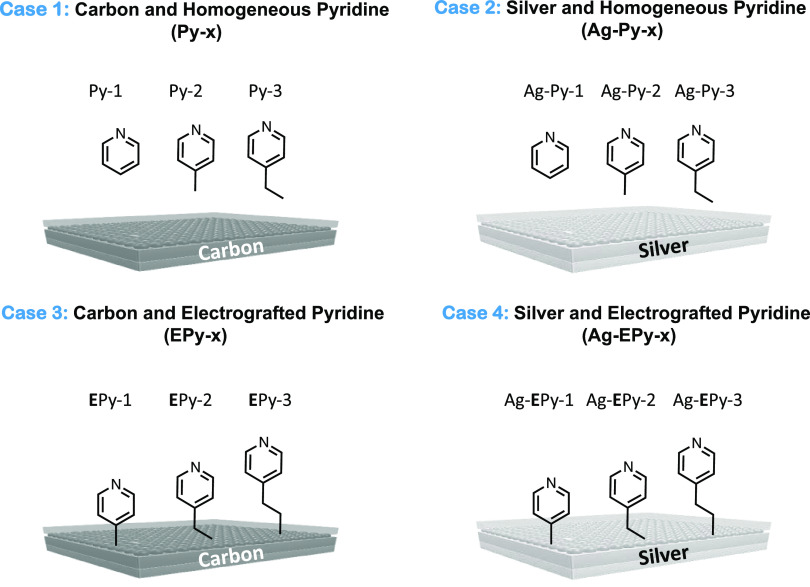
Schematic of case 1: Homogeneous pyridine molecular catalysts with glassy carbon electrodes (GCE) (Py-1, Py-2, and Py-3); case 2: homogeneous pyridine molecular catalysts with silver electrode catalysts (Ag-Py-1, Ag-Py-2, and Ag-Py-3); case 3: heterogeneous electrografted pyridine catalysts onto glassy carbon electrodes (EPy-1, EPy-2, EPy-3); and case 4: heterogeneous electrografted pyridine catalysts onto silver electrodes (Ag-EPy-1, Ag-EPy-2, and Ag-EPy-3).

Here, the immobilization of pyridine derivatives onto Ag nanoparticles was demonstrated using a molecular electrografting technique that incorporates diazonium chemistry, enabling a one-pot and irreversible fixation onto the electrode surface, resulting in reduced overpotentials versus the individual catalysts. We investigate the CO_2_RR performance for a variety of hybrid molecular/nanoparticle catalysts, showing how the distance between the pyridine capture site and the electrode surface impacts the overall catalytic efficiency. To examine the propensity for a combined transition-metal and molecular catalyst to work in unison, we designed a set of experiments to test both homogeneous and heterogeneous catalytic systems (Figure 1). Namely, we tested the activity and selectivity for CO_2_ electroreduction in four control cases: using a carbon and silver electrode with pyridine present only in the electrolyte (cases 1 and 2, respectively), pyridine electrografted to a carbon electrode (case 3—EPy), and pyridine electrografted to a silver electrode (case 4—Ag-EPy). Before we could make these comparisons, however, we first needed to confirm that it was indeed possible to irreversibly affix pyridine molecules to silver catalysts.

While all combined catalysts showed increased activity, the 2-carbon chain length pyridine compound elicited a 200 mV decrease in the onset potential at 1 mA/cm^2^ and a 10-fold improvement versus bare Ag at a voltage of −0.7 V vs reversible hydrogen electrode (RHE). We provide attenuated total reflectance surface-enhanced infrared absorption spectroscopy (ATR-SEIRAS) measured and density functional theory (DFT) computations of the system to assess the production rates and mechanisms of different catalysts. Finally, we demonstrate the stability and efficiency of the electrografted system at elevated current densities through flow cell experiments up to 200 mA/cm^2^.

## Results and Discussion

### Synthesis and Structural Characterizations of Electrografted Pyridines onto Electrode Surfaces

**Figure 2:**
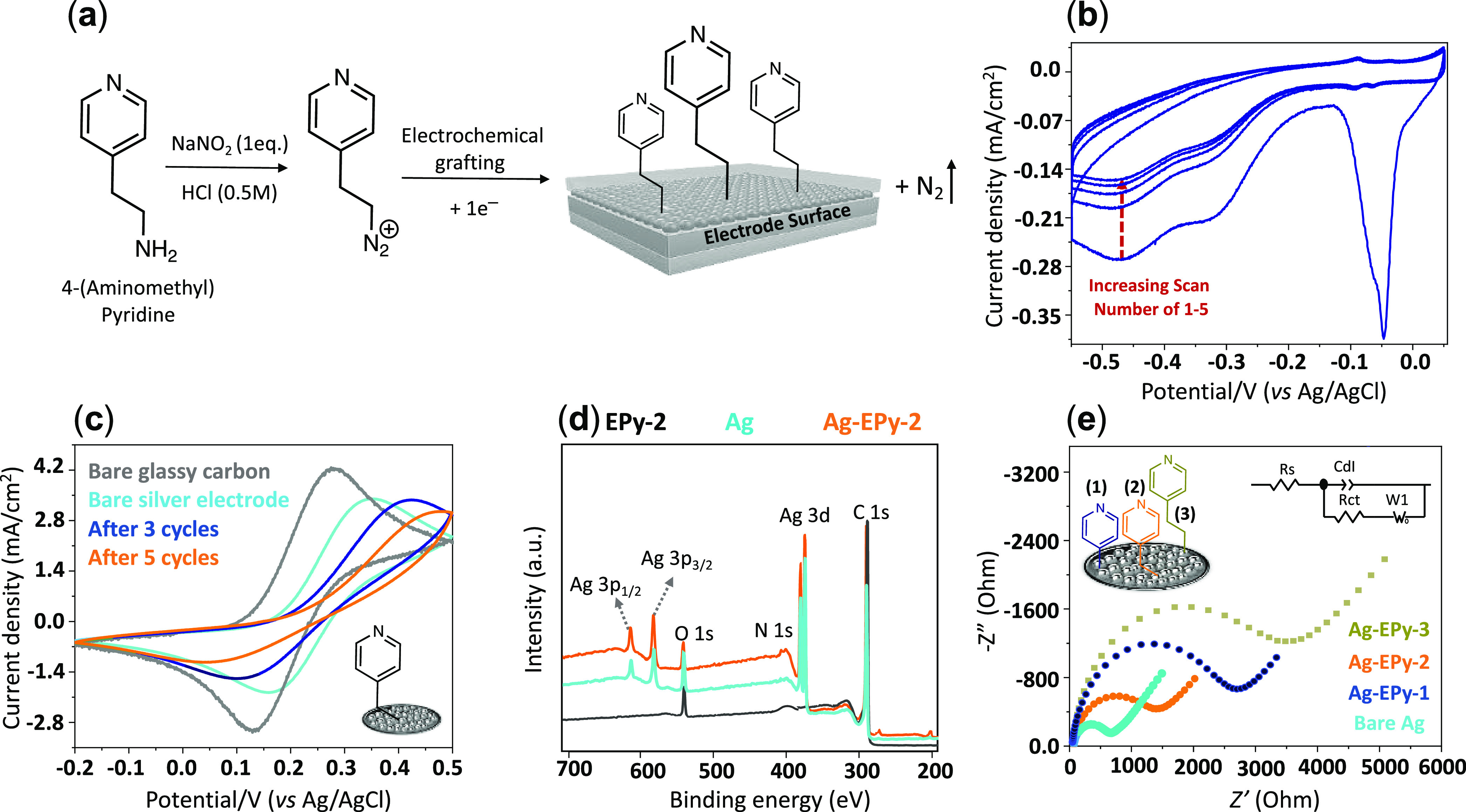
(a) Preparation of pyridine–diazonium cations generated *in situ* to form electrografted pyridines; (b) electrografting voltammogram of 5 mM of Py-2 onto a silver electrode in 2 mM NaNO_2_ and 0.5 M HCl at a scan rate of 50 mV/s; (c) cyclic voltammetry (CV) comparison of a ferrocyanide redox probe (2.5 mM K_4_Fe(CN)_6_/200 mM KNO_3_) before and after electrografting at a scan rate of 50 mV/s; (d) X-ray photoelectron spectroscopy (XPS) survey spectra of Ag, EPy-2, and Ag-EPy-2; and (e) Nyquist diagrams of a bare glassy carbon, Ag, and Ag-EPy-*x* in 2.5 mM and 200 mM KNO_3_.

In line with the motivation for controlling the distance between the pyridine ring and the Ag surface, we used pyridines of different carbon chains, denoted as Ag-EPy-1, Ag-EPy-2, and Ag-EPy-3. To begin the electrografting process in H-cells, we first prepared the Ag electrode using electrodeposition of a 1 mM AgNO_3_ solution with 0.1 mM NaHCO_3_ at a constant applied potential of −0.2 V vs RHE for 200 s (Figure S1).^48^ With the Ag surface ready, aminopyridine derivatives were then covalently immobilized on the silver surface through diazotation using NaNO_2_ (2 mM) in HCl (0.5 M) solution to form pyridines-N_2_^+^ (Figure 2a).^47,49^ Once the diazotation reaction was completed, the diazonium cation was reduced on the electrode surface *in situ* between −0.5 and 0.05 V vs Ag/AgCl using five cyclic voltammetry (CV) cycles at a scan rate of 50 mV/s shown in Figure 2b.^24,50^ A characteristic irreversible reduction peak was used to identify the reduction of diazonium salt to form the aryl radical intermediate, promptly followed by the formation of a covalent bond to the electrode surface and the release of N_2_ gas.^51−54^ During the 2nd to 5th cycles, the cathodic peak shifted to more negative potentials and lower current densities, indicating successful electrografting of pyridine on the electrode surface.^49^ An extra peak at ∼−0.32 V vs Ag/AgCl shown in Figure 2b arises from the reduction of Ag^+^ to Ag^0^,^55^ whereas the sharp peak at ∼−0.04 V vs Ag/AgCl indicates complete AgO reduction to metallic silver,^56^ which is absent in EPy-2 (Figure S2b).

A CV study with a ferrocyanide redox probe (aqueous 2.5 mM K_4_Fe(CN)_6_/200 mM KNO_3_) better illustrates the successful formation of the organic layer on the electrode surface. For this purpose, the second CV cycle of each individual catalyst was compared and is demonstrated in Figures 2c and S2b. A significant decrease in the current density of the probe’s redox profile was observed before and after molecular deposition, indicating that the access of the probe to the electrode is effectively obstructed due to the formation of the pyridine layer on the electrode surface.^49^ To further confirm the presence of surface pyridine, we performed a surface analysis with X-ray photoelectron spectroscopy (XPS) (Figure 2d).^57^ Survey spectra of EPy-2, Ag-EPy-2, and bare Ag electrode find C 1s, N 1s, and O 1s peaks at 284, 399, and 530 eV, respectively. Peaks at 368, 573, and 604 eV correspond to Ag 3d, 3p_3/2_, and Ag 3p_1/2_, correspondingly.^58^ The results are in agreement with similar previous reports.^59,60^ As expected, no Ag 3p peaks were observed in the case of EPy-2. The peak at 399.5 eV in EPy-2 had a very slight shift to 399.7 eV in the case of Ag-EPy-2, indicating an electrostatic interaction between the silver surface and pyridine groups (Figure S3b). The small O 1s peak seen in all cases is attributed to the spontaneous oxidation of the carbon surface when exposed to air (Figure S3).^61^

Lastly, to assess the charge-transfer dynamics of the affixed pyridine at the electrode’s surface versus bare carbon and silver, we used electrochemical impedance spectroscopy (EIS) in the 2.5 mM [Fe(CN)_6_]^3–/4–^ redox probe in 200 mM KNO_3_ solution (Figures 2e and S2c). The Nyquist plots pictured in Figure 2e ascribe the largest charge transfer with Ag-EPy-3 (3510.2 ± 72.1 Ω) followed by Ag-EPy-1 (2534.4 ± 50.3 Ω), Ag-EPy-2 (1492.6 ± 38.9 Ω), and bare Ag (690.7 ± 21.9 Ω). A significant increase in the resistance can be seen across all systems in the presence of the immobilized pyridine compounds.^62^

### Electroreduction of CO_2_ in H-Cell

**Figure 3:**
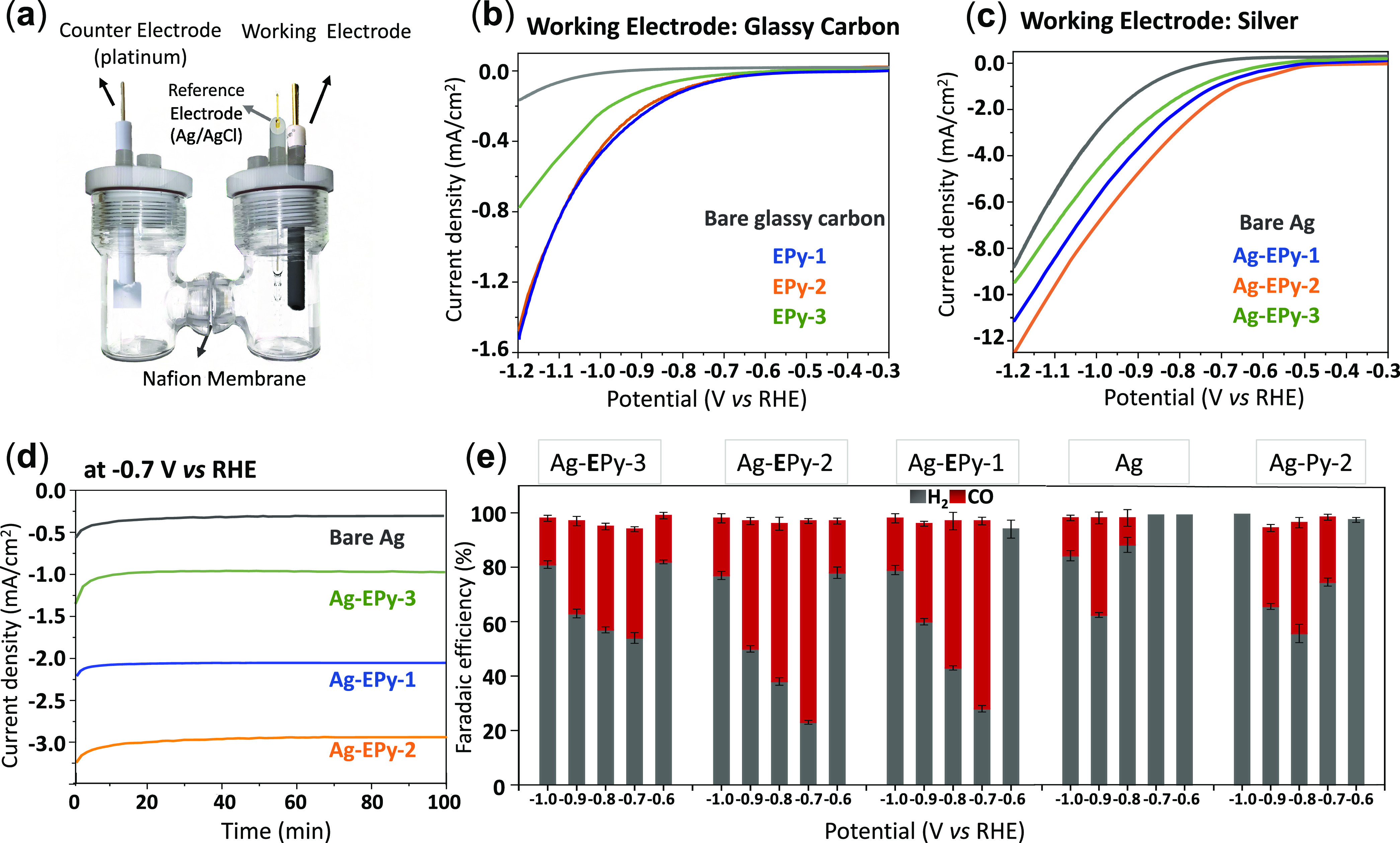
(a) Schematic for the H-cell setup. Linear sweep voltammetry (LSV) comparison of heterogeneous pyridine electrocatalysts at (b) a glassy carbon electrode (EPy-*x*) and (c) a silver electrode (Ag-EPy-*x*) under CO_2_. (d) Chronoamperometry comparison of bare Ag and Ag-EPy-*x* in 0.1 M KHCO_3_ at −0.7 V vs RHE under CO_2_. (e) Faradic efficiency (FE) comparison of Ag-EPy-*x*, bare Ag, and homogeneous Ag-Py-2 at −0.6, −0.7, −0.8, −0.9, and −1.0 V vs RHE in 0.1 M KHCO_3_.

With the combined silver nanoparticles and electrografted pyridine molecular catalysts formed, we now test the comparative CO_2_ electrolysis performance of the varied chain lengths (Ag-EPy-*x*), as well as the controlled systems. These were first performed in a two-compartment H-cell with a three-electrode configuration including a Ag/AgCl reference electrode (RE) and a Pt counter electrode (CE) in a CO_2_-saturated 0.1 M KHCO_3_ aqueous solution (Figure 3a).^63^

The initial CV comparison of the heterogeneous systems (cases 3 and 4) was carried out in the presence and absence of CO_2_. The comparison highlights a substantial increase in the current density upon saturation of the solution with CO_2_ (Figures S4 and S5). The linear sweep voltammetry (LSV) of the immobilized pyridine catalysts (EPy-*x*) onto a glassy carbon electrode (GCE) sees a CO_2_ reduction onset potential arise at ∼−0.8 V vs RHE (Figure 3b). Compared to that of the GCE control, the Ag-EPy-*x* electrode systems show a general increase in current density coupled with a noticeable positive shift to lower onset potential (∼−0.6 V vs RHE), defined at 1 mA/cm^2^ (Figure 3c). A large overpotential is often attributed to the activation energy barrier of the initial electron transfer that forms the CO_2_^•–^ intermediate, which is poorly stabilized by the silver and glassy carbon electrode surfaces.^64^ It is evident that the immobilized pyridine molecules show a clearly more positive onset potential compared to bare Ag, highlighting the role of the affixed pyridine as a cocatalyst, which improves the overall electrochemical activity as compared to the bare Ag electrode alone.

Electrolysis was then performed at fixed potentials to allow for the comparative activity and selectivity to be measured (Figures S6 and S7). In all cases, H_2_ and CO were the only gaseous products measured and formate was observed as the sole liquid product. The resulting chronoamperometry measurements of Ag-EPy-*x* and bare Ag at −0.7 V vs RHE in Figure 3d show that the highest current density is observed for Ag-EPy-2, with an ∼10-fold increase in current density over the bare Ag sample at the same potential. The EPy-*x* showed low catalytic performance toward CO_2_RR (Figure S8). The increase in activity is also exhibited as improved selectivity toward CO, as shown in Figure 3e, where the combined transition metal and molecular catalysts exhibited higher CO_2_-to-CO selectivity than both the heterogeneous Ag catalyst (Ag-Epy-2) and the homogeneous pyridine catalyst (Ag-Py-2) over all potentials. These results highlight not only the advantages of the combined catalytic system (case 4) versus the separate cases (cases 1–3) but also the importance of the pyridine chain length. For example, Ag-EPy-2 showed the greatest catalytic activity in the heterogeneous media at −0.7 V vs RHE (Figure 3e), with the FE = 74% and *j* = −3.1 mA/cm^2^ compared to Ag-EPy-1 (FE: 69%; *j*: −2.26 mA/cm^2^) and Ag-EPy-3 (FE: 40%; *j*: −1.2 mA/cm^2^).

For a systematic comparison, 5 mM of homogeneous pyridine catalysts Py-x (Figures S9–S17) in 0.1 M KHCO_3_ was also applied over the same potential range of −0.5 to −1.0 V vs RHE. These homogeneous catalysts showed a low catalytic activity at a higher potential of ∼−0.8 V vs RHE in comparison to the heterogeneous controls.

The above electrochemical experiments highlight the potential for increased activity and selectivity for the combined transition metal and molecular system. Next, we apply mechanistic studies to investigate the interactions between the Ag support, the electrografted pyridine, and the CO_2_ reduction reaction.

### Mechanistic Study

The individual role of pyridine as well as electrode activity toward electrochemical CO_2_RR needs to be considered for the mechanistic study. The activity of pyridines may be related to the formation of a pyridinium radical,^41^ which acts as a one-electron charge-transfer mediator to form PyCOOH^0^.^26,65−67^ Considering the reaction intermediates,^68−70^ the main cocatalytic functionality of pyridinium could be to facilitate proton-coupled electron transfer (PCET) or proton-coupled hydride transfer (PCHT).^21,70^ It should be noted that the pyridinium film formed on the electrode surface can increase the local pH.^44^ Fang et al.^38^ observed that the immobilized 4-pyridinylethanemercaptan on a Au electrode can improve selectivity by facilitating a proton transfer to the key intermediate of HCOO*. This reaction proved to be pH-dependent, where, at lower pH (pH ≤ 6.5), they observed the reduction of pyridinium through a one-electron-transfer process followed by generation of dihydrogen and pyridine. Shaw’s group investigated a possible pyrH^+^ activity at pH = 9.2 to pH = 5.0 using a Au working electrode (WE).^35^

On the other hand, the role of the transition-metal electrode support in CO_2_RR influences the nature of the formed product. Hori’s group has extensively studied CO_2_ reduction on noble metals and reports the main product of gold (Au) electrodes to be CO, while that for platinum (Pt) electrodes is H_2_.^71^*N*-Arylpyridinium salts have been shown to act as precatalysts and increase the selectivity of C2 products on copper electrodes.^36^ It should be noted that when bound to the electrode surface through either a surface bond or while chemically adsorbed, pyridine and pyridinium are chemically inert to the CO_2_RR process due to their strong interaction with the electrode surface through their −*N*-catalytic active site.^45^

In the current work, we performed a number of mechanistic studies to better understand (i) the role of pyridine molecules in facilitating the electron transfer and improving the reaction rate; (ii) the role of pyridine as a cocatalyst in capturing CO_2_ and enhancing mass transport; and (iii) the relationship between different carbon chains and the synergy between molecular pyridines and the electrode surface. These studies include CV sweeps under different scan rates, measurements of the Tafel slopes of different catalysts, a DFT analysis, and operando ATR-SEIRAS spectroscopy.

The difference in peak separation upon applying the CV scan rate can be used to quantify the heterogeneous electron-transfer rate constant between the electrode and molecular catalyst species.^72^ Therefore, to shed light on the increased rate of electron transfer between the electrode and the catalytic layer with different carbon chains, the detailed electroreduction of CO_2_ in both EPy-*x* and Ag-EPy-*x* was evaluated at several scan rates of 20, 40, 60, 70, 100, 200, 300, 400, and 500 mV/s and a linear relationship between the reduction peak currents and scan rate was observed (Figure S19). The experimentally determined slopes^73−76^ were applied, and the ECSA of GCE (0.002 cm^2^), EPy-1 (0.002 cm^2^), EPy-2 (0.003 cm^2^), EPy-3 (0.002 cm^2^), Ag (0.006 cm^2^), Ag-EPy-1 (0.026 cm^2^), Ag-EPy-2 (0.036 cm^2^), and Ag-EPy-3 (0.027 cm^2^) was calculated accordingly. Comparing the results in Figure S19, the higher ECSA of Py-2 in both cases of GCE and Ag electrodes again confirms the highest catalytic performance of the EPy-2 and Ag-EPy-2 among the others, indicating a higher rate of electron transfer.

Next, to obtain additional insight into the reaction kinetics of the best catalyst, Tafel slopes of EPy-*x* and Ag-EPy-*x* were calculated in 0.1 M KHCO_3_ for the electrochemical CO_2_RR (Figure S20). They are 124.2, 120.6, and 152.2 mV/dec for Ag-EPy-1, Ag-EPy-1, and Ag-EPy-3, respectively. The same trend was observed in the case of EPy-*x*. The smallest Tafel slope value belonging to EPy-2 and Ag-EPy-2 confirms that the faster reaction kinetics are influenced by better electron transfer between the molecular catalyst and silver electrode surface.^77^ Similar behavior has been observed previously using *N*-based compounds.^78−80^ For example, Zhao et al.^81^ highlighted the importance of amine molecular catalysts with tunable alkyl chains toward CO_2_RR in the presence of Au nanoparticles.

**Figure 4:**
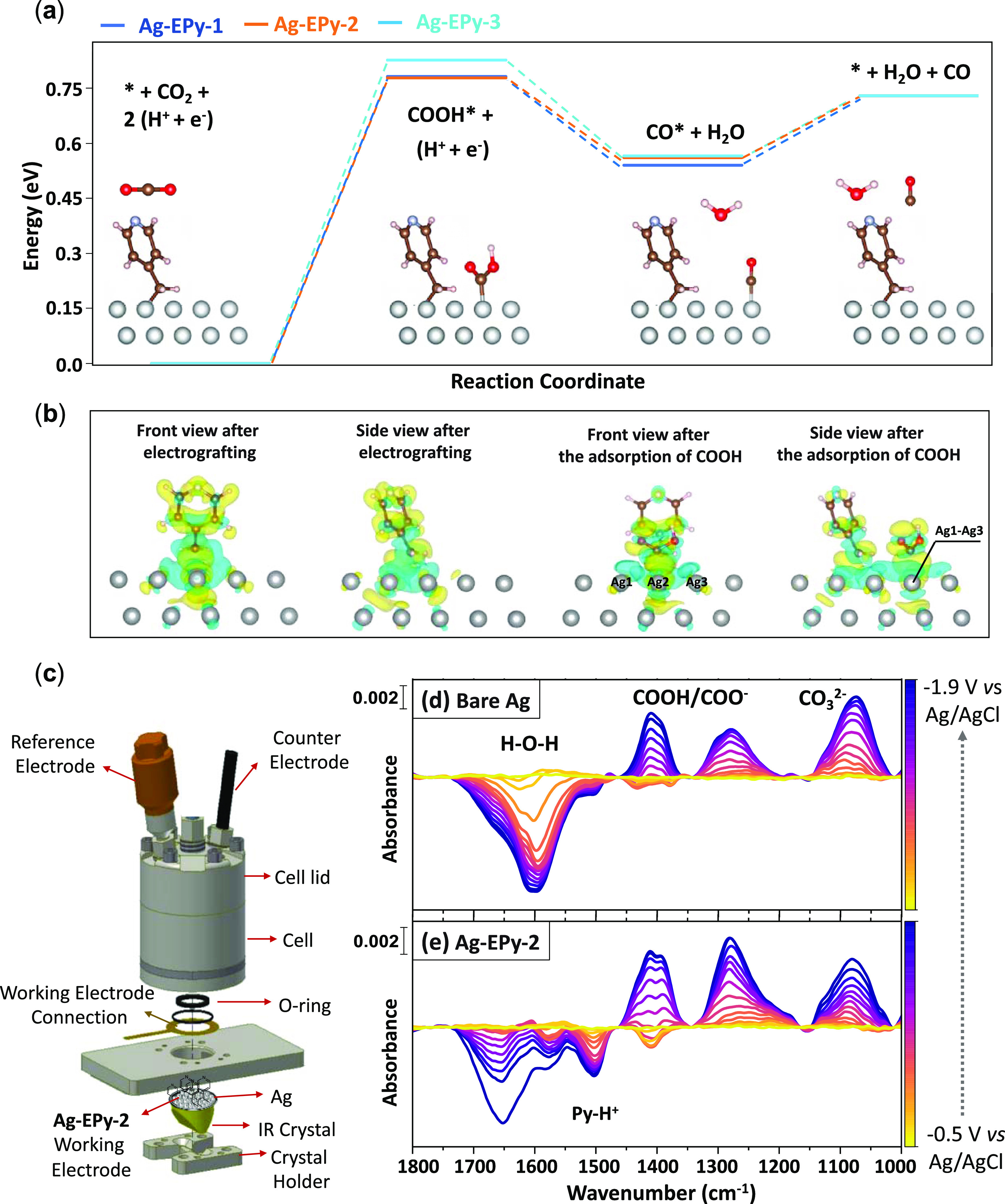
(a) Reaction energy vs reaction coordinate (schematics shown for Ag-EPy-2). (b) Deviations in charge densities after electrografting Ag-EPy-2 from different views before and after *COOH adsorption. (c) Schematic for the customized attenuated total reflectance surface-enhanced infrared absorption spectroscopy (ATR-SEIRAS). (d, e) ATR-SEIRA transmission spectra of immobilized Ag-EPy-2 at different potentials in a 0.1 M KCl electrolyte under CO_2_.

DFT calculations were performed to gain further insight into the increased CO_2_RR activity of the deposited pyridine catalysts with the silver electrode (Ag-EPy-*x*) (Figures S21–S24). The role of the pyridine group and that of the length of the carbon chains in facilitating the electron transfer toward the improvement of the CO_2_RR catalytic performance are observed by calculating the reaction energy diagram and charge delocalization. CO_2_RR to CO is studied through two PCET steps^82^ and via carboxyl intermediate formation. As depicted in Figure 4a and Tables S1 and S2, the Ag-EPy-2 demonstrates the lowest energy barrier compared to the other two Ag-EPy-*x*. This reaction energy diagram reveals that the adsorption of *COOH is the rate-determining step, where different Ag-EPy-*x* demonstrate different energy barriers for this step through the following order: Ag-EPy-2 (779 meV) < Ag-EPy-1 (784 meV) < bare Ag (804 meV) < Ag-EPy-3 (828 meV) (Table S3). This order is consistent with the experimental observation of the overpotentials.

To further investigate the role of pyridines in this elementary step, charge delocalization around the *N* group of the pyridines and active Ag sites is demonstrated in Figures 4b, S25, and S26. The Bader charge analysis is performed to quantify the charge that is transferred during the reaction. According to Figure S27, *COOH is adsorbed in a top-site configuration (e.g., Ag 2 in Figure 4b, with adjacent Ag1 and Ag3 atoms). Results of the Bader charge analysis and charges attributed to each Ag atom, before and after the *COOH adsorption, reveal that the largest charge donation occurs with Ag-EPy-2 (0.1783 e^–^) followed by Ag-EPy-1 (0.1755 e^–^) and Ag-EPy-3 (0.1671 e^–^) (Table S4). The largest contribution comes from the central Ag site (Ag 2) in all three cases, which is anticipated because of its shorter distance from the adsorbate. Two oxygen atoms within the adsorbed carboxyl intermediate change the electron density around Ag1 and Ag3. However, the oxygen above the Ag3 is bonded to the hydrogen, lowering its electronegativity compared to the other oxygen atom above the Ag1; thereby, Ag1 donates more charges than Ag3.

The reaction pathways for the electroreduction of CO_2_ to CO and formate using the Ag-EPy-2 electrode were further studied using *in situ* ATR-SEIRAS (Figures S28–S30), which aids in characterizing the catalytic active sites experimentally. To highlight the role of electrografted pyridines toward CO_2_RR, the results were compared with those of the bare Ag.^32,83^ The measurements were performed in a customized, spectroelectrochemical H-cell, which housed the Ag-EPy-2 working electrode, a Ag/AgCl reference electrode, and a graphite counter electrode (Figure 4c).

Recording the evolution of the ATR spectra on the bare Ag and Ag-EPy-2 over time at increasing potentials from −0.5 to −1.9 V vs Ag/AgCl in 0.1 M KCl saturated with CO_2_, we can gain insight into the reaction mechanism and product intermediates. As shown in Figure S30, after several minutes of electrolysis, the CO band arises at ∼1980 cm^–1^. Two large peaks at 1288 and 1389 cm^–1^ correspond to the *COOH and a symmetric stretch of COO^–^.^84,85^ It should be noted that CO_2_ in water is a source of Brønsted acids (*CO_3_H_2_, *CO_3_H^–^). The peak at ∼1501 cm^–1^ belongs to pyridinium ions resulting from Py protonation on Brønsted acid sites (CO_3_H_2_, CO_3_H^–^) formed from the reaction of CO_2_ in water,^86,87^ which can be converted to the pyridinium radical–CO_2_ under electrochemical conditions to form a carbamic zwitterion.^26^ Additional bands situated between 1000 and 1450 cm^–1^ correspond to interfacial carbonates and bicarbonates and show similar trends in both cases, pointing to comparable surface pH values.^88^ The shoulder peak at ∼1575 attributed to Py is absent in the case of bare Ag. In the case of Ag-EPy-2, a clear increase in the intensity of *COO^–^ compared to that of bare Ag was observed, which highlights the important role of pyridine as the dual active site in the capture and potential reduction of CO_2_. The peak at 1670 cm^–1^ belongs to a combination of the H–O–H bend and C=O asymmetric stretch assigned to the *COOH/*COO^–^ intermediates, which is in agreement with previous reports.^89^

The increase in the intensity of CO_2_ consumption at ∼2400 cm^–1^ shows that CO_2_ is consumed at a [3:2] ratio for [Ag-EPy-2: bare Ag] (Figure S30). The initial shift to a higher wavenumber in the CO_2_ peak may be attributed to the increasing coverage of CO_2_ on pyridine-modified surfaces, whereas the same shift is negligible in the case of bare Ag.^90^ Based on previous reports^91−93^ and our findings, we hypothesize that the pyridine molecules have a distinctive role as cocatalysts in promoting the electrocatalytic activity of the silver electrode, responsible for a superior catalytic performance.

With the experimental demonstration of the combined catalytic system resulting in improved electrochemical performance and potential mechanisms investigated, we aimed to apply the combined system in a flow cell operation to reach higher current densities.

### Electrografting of Pyridines onto Gas Diffusion Electrodes (GDE) and Electroreduction of CO_2_ in Flow Cells

Although H-cells are useful for exploring material combinations and mechanistic studies with fine control, the performance of catalysts within H-cell aqueous systems for CO_2_ reduction is limited by the low solubility of CO_2_ in aqueous solution and accessibility of active sites.^94,95^ For elevated reaction rates due to improved mass transport and higher surface areas, researchers have turned to flow cell systems, which may use a gas diffusion electrode to support catalytic structures.^44,49−53,96−99^ Here, we sought to demonstrate the implementation of the Ag-EPy system onto a gas diffusion layer (GDL) and in different flow cells to prove both the catalytic stability of the system and demonstrate the potential for two catalytic systems to be combined in this setup.

**Figure 5:**
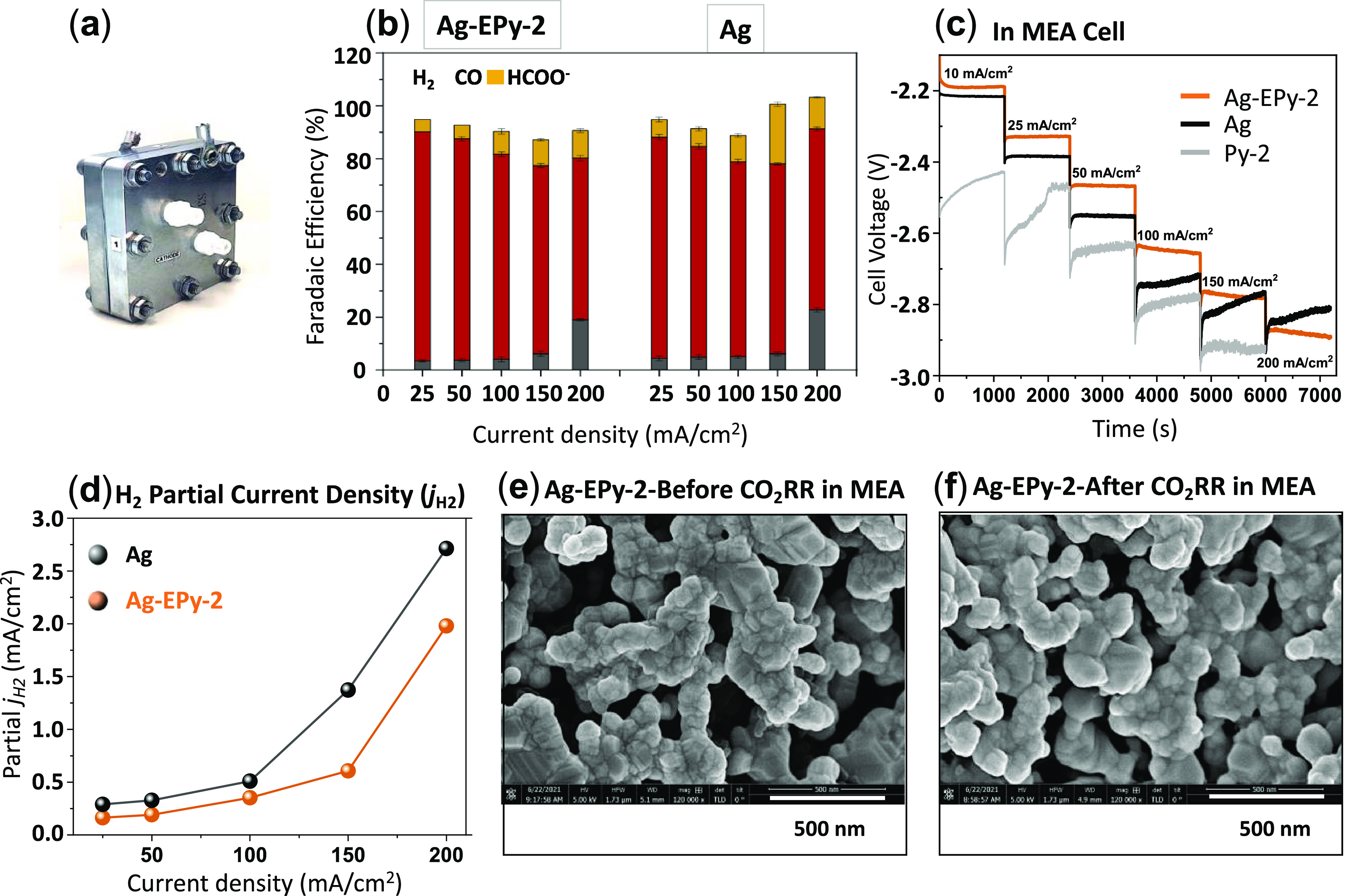
(a) Membrane electrode assembly (MEA) cell for the electrochemical reduction of CO_2_; (b) faradic efficiency (FE) comparison of Ag and Ag-EPy-2 at current densities of 25, 50, 100, 150, and 200 mA/cm^2^ in an MEA; (c) sketch graph of voltage against time at different current steps in the range of 25–200 mA/cm^2^; (d) partial current density comparison of hydrogen (*j*_H2_) using Ag and Ag-EPy-2 for CO_2_ electroreduction; scanning electron microscopy (SEM) of Ag-EPy-2 (e) before and (f) after electrochemical CO_2_RR.

To this end, we chose the best catalyst, Ag-EPy-2, to be translated from the H-cell to zero-gap membrane electrode assembly (MEA). The MEA cell consists of an anode chamber with a liquid phase anolyte and a cathode chamber with a gas phase inlet (Figures 5a, S31, and S32).^100,101^ In this design, the humidified CO_2_ is delivered directly to the active materials through a serpentine flow channel located at the back side of the gas diffusion electrode (GDE). For the flow cell configuration (Figures S33 and S34), the catholyte solution was circulated between the GDE and the membrane.

The GDE was prepared by sputtering 10 nm Ag onto the gas diffusion layer (GDL) to form a hydrophobic and microporous layer. Next, Py-2 was successfully electrografted onto the Ag using a technique identical to that described above to form Ag-EPy-2 (Figure S35). The electrografting was conducted with 3, 5, and 10 cycles to determine the best surface coverage with pyridines for the CO_2_RR and ensure the pyridine fully coated the now three-dimensional (3D) porous electrode structure. Similar to H-cell, the five cycles demonstrated the best catalytic activity, while after five cycles, the electrode conductivity decreased. Immobilized Ag-EPy-2 on a GDE was then used as a working electrode with a nickel counter electrode as the anode, both with a surface area of 6 cm^2^. To the best of our knowledge, it is the first report on molecular electrografting immobilization on GDE.

The catalytic activity of Ag and Ag-EPy-2 was subsequently investigated at current densities ranging from 25 to 200 mA/cm^2^ in an MEA cell (Figure 5b). To determine the cell potential at each current density, currents were applied stepwise (Figure 5c). Comparing Ag, EPy-2, and Ag-EPy-2 finds a lower cell potential when Ag-EPy-2 is used, highlighting the importance of having both Ag and pyridine in combination for enhancing the overall catalytic performance, which has been hypothesized in this work. The pyridine catalyst also showed high CO selectivities, on par with that of pure Ag in an MEA configuration, with slightly lower observed HER, which may be attributable to the reduced operating voltages and earlier onset potential of CO versus bare Ag (Figure 5d). The reduction in applied potential then indicates that the complex, and the approach in general, can show CO_2_RR advantages even when the selectivity of the base system is high owing to the successful design of the Ag-EPy-*x* system.

To further study the role of electrografted pyridine in improving the silver electrode surface stability, similar experiments were performed using a flowing catholyte system rather than the above MEA configuration (Figure S36).^102^ Increasing the product selectivity along with increasing the current density in Ag-EPy-2 compared to that in bare Ag shows the increasing stability of the Ag surface after electrografting. Although the overall selectivity toward CO was lower, it was maintained at higher overall current densities. Further optimization of these systems may then be needed to avoid HER.

Finally, we performed scanning electron microscopy (SEM) to visualize the surface morphology and document any morphological stability changes during the CO_2_RR of the Ag-EPy system. A comparison of SEM before and after 2 h of CO_2_ electrolysis in the flow cell finds no significant variations in surface morphology, attesting to the high stability of the catalysts to the local environment imposed at high current density (Figures 5e,f and S37). This is further confirmed by atomic force microscopy (AFM) (Figures S38 and S39) and XPS (Figure S40) studies. These studies including height sensor images, peak force error images, and 3D topographies show no discernible change in catalyst microstructure during the high-electrolysis process.

It should be noted that the increased formate selectivity in GDEs (flow cells and MEA) can be attributed to the higher alkalinity around the catalyst surface (pH > 12). Previous studies have also reported similar trends where an increase in formate and a drop in CO selectivity are seen under extreme alkaline conditions.^103^ The selectivity switch from CO to formate has been attributed to the reduced ability of hydronium to assist in the first protonation step of the *COOH intermediate.^104^

Herein, we report a new approach to improve CO_2_RR through the design of a hybrid molecule/support structure. The approach taken in this work is built on the synergistic effect of pyridine groups with a Ag surface, which was carefully investigated by altering the alkyl chain length of several pyridine derivations. In successfully electrografting pyridine molecules to a Ag electrode, additional capturing sites and favorable binding interactions were created, contributing to an increase in the overall catalytic performance. We have shown that electrografted pyridine compounds enhance the stability of the key carboxyl intermediate (*COOH) and thereby lower the reaction energy barrier for the rate-determining step and facilitate the CO_2_RR. Four considerations contributed to the adoption of pyridine as a promoter: (i) chemically anchoring pyridine groups on the electrode surface through diazonium chemistry can provide an opportunity for the nitrogen atom to be coordinated with *COOH; (ii) use of heterogeneous media requires smaller molecular loading to achieve the desired catalytic effect; (iii) creating optimal synergy between pyridines and the electrode surface to promote charge transfer and facilitate CO_2_RR; and (iv) increasing mass transport to the electrode interface using pyridine groups with CO_2_-capturing capabilities. The former modulates the electronic structure of the active Ag sites through an optimum charge delocalization for Ag-EPy-2, Ag-EPy-1, and Ag-EPy-3, respectively.

## Conclusions

In summary, simple and inexpensive pyridine molecules were shown to efficiently catalyze the electroreduction of CO_2_ to C1 products with high selectivity and current density at low potential. Several pyridines of varying alkyl chain lengths were studied to better understand the synergistic effect between the catalyst structure and the surface electrode. Combined, we demonstrated the catalytic advantages greater than that of the individual catalyst structures, with a reduction in onset potential allowing for improved selectivities in H-cell studies. We hope that the above approach demonstrates the potential for this strategy to be generalized for other transition metals and with further intentionally designed molecular catalysts to continue to improve the efficiency of CO_2_RR systems.

## Experimental Section

### Reagents and Chemicals

All reagents and solvents were of commercial reagent grade and were used without further purification, except where noted. Reagents not listed were purchased from Sigma-Aldrich. 4-(2-Aminomethyl) pyridine (98%), 4-methylpyridine (99%), 4-propylpyridine, 4-aminopyridine (98%), potassium ferrocyanide(III) (99%), sodium nitrite (97%), silver nitrite (99%), deuterium oxide (D_2_O), (>99.8% D), and potassium bicarbonate (99.7%) were purchased from Sigma-Aldrich Company. All aqueous solutions were prepared using Millipore water (18.2 MΩ cm). The glassy carbon surface was polished with 1, 0.3, and 0.05 μm alumina slurries. The electrodes were then ultrasonicated in acetone, ethanol, and water.

### Materials and Characterizations

All of the spectroscopy data for structural characterizations were obtained using the research facilities at Delft University of Technology. ^1^H NMR chemical shifts (δ) were reported in ppm in deuterium oxide (D_2_O). The NMR data was processed in MestReNova software. The reduced products observed in the cathodic compartment were periodically collected from the reaction headspace and tested by gas chromatography (GC). The concentration of gaseous products (CO and H_2_) was obtained from GC, and the average of four injections was used to calculate their faradic efficiencies. The gas product from carbon dioxide (CO_2_) electroreduction (CO, H_2_) was analyzed using chromatograph (InterScience PerkinElmer Clarus 680) coupled with two thermal conductivity detectors (TCDs) and a flame ionization detector (FID).

The gaseous products (here denoted as “*i*”) were quantified according to the formula eq 1
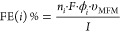
1where “*n*_*i*_” is the number of the electrons needed for CO_2_ reduction to product *i*, “*F*” is the Faraday constant, “ϕ_*i*_” is the volume fraction of the gases, *i* is the current measured at the time of the injection, and “υ_MFM_” is the molar gas flow rate measured by mass flow meter at the cell outlet and corrected according to the product mixture. The volume fraction of the gases is calculated by calibrating the GC using a diluted mixture of gas bottles with known concentrations.

The gas outlet of the electrolysis cell (either H-cell or flow cell assembly) was connected to the sampling port of the GC, which injects a certain volume of the prechamber filled with a saturated product gas from CO_2_ electrolysis. An injection loop takes around 5 min including the back-flush and stabilization time. A Molsieve-S4 column connected to a thermal conductivity detector (TCD) was used to analyze hydrogen (H_2_) and carbon monoxide (CO) gases. The peak position and calibration line for CO and H_2_ are shown in Figures S42 and S43.

The electrical energy spent at the end of electrolysis must be equivalent to the chemical energy of formation of a compound *i* according to the formula eq 2
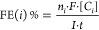
2where *n*_*i*_ is the number of electrons to be exchanged to produce *i*, Faraday’s constant (96,485.3 C/mol e^–^) is the charge in Coulombs for one mole of electrons as *F*, the concentration of *i* specie in mole is denoted [*C*_*i*_]. The net current flux across the electrical circuit is in Amperes shown with *i*, and “*t*” is the total time in seconds. In our test, formate was the primary liquid product as identified by high-performance liquid chromatography (HPLC, Agilent 1200 HPLC using an Agilent HiPlex-H column 300 × 7.7 mm with 20 mM H_2_SO_4_ as mobile phase at 0.6 mL/min rate).

X-ray photoemission spectroscopy (XPS) measurements were performed with a Thermo Scientific K-Alpha spectrometer using a monochromatic Al Kα excitation source. The spectrometer was calibrated using the C 1s adventitious carbon with a binding energy of 284.8 eV. The base pressure at the analysis chamber was about 2 × 10^–9^ mbar. The spectra were recorded using a spot size of 400 μm at a pass energy of 50 eV and a step size of 0.1 eV. Scanning electron microscopy (SEM) measurements were carried out with an FEI NovaNano SEM using secondary electron imaging with immersion lens mode and a 5 kV electron acceleration voltage.

Atomic force microscopy (AFM) was applied to characterize the surface microstructure of the silver catalyst layer of the gas diffusion electrode. The Bruker’s dimension icon equipped with TESPA-V2 tip performed the AFM characterization in a soft tapping mode. The height sensor and peak force error images of the catalyst layer were obtained during the test, and the 3D images were constructed based on the high sensor data by the NanoScope Analysis software.

### Preparation of the Gas Diffusion Electrode

Ag-GDEs were made by magnetron sputtering (AJA International Inc.) Ag (MaTeck Germany, 99.9% purity) onto Freudenberg H14C10 GDL (Fuel Cell Store) to obtain a thin film of Ag with 10 nm and 100 nominal thicknesses. During sputtering, the power supply was kept at 50 W DC with an Ar flow at 20 sccm (standard cubic centimeters per minute). The geometrical area of the GDL was 2.25 and 6.25 cm^2^ for the GDE-type and MEA-type flow cells, respectively. The electrode samples were kept in an argon-filled glovebox prior to the electrografting and/or electrochemical testing.

### Thin-Film Cathode Preparation for ATR-SEIRAS

Thin-film cathodes were deposited on 60° Ge ATR crystals (Pike Technologies, 013-3132). These crystals were polished using alumina powder suspensions of decreasing grain sizes (1.0, 0.3, and 0.05 μm) and then sonicated for 5 min in iso-propyl alcohol and deionized water. Before mounting in the DC magnetron sputtering setup, crystals were wiped with acetone using cotton swabs. Deposition of the Ag catalyst layer was performed in a magnetron sputtering system (PREVAC Project 229) at a chamber pressure of 25 μbar, an argon flow rate of 15 sccm, and a power rate of 25 W for a deposition rate between 0.013 and 0.014 nm/s and a thickness of 40 nm. The presence of the catalyst was confirmed both optically and by measuring the resistance over the film using a multimeter, which was between 3 and 4 Ω. This procedure is strongly based on that reported in the previous literature but avoids air- or argon-plasma cleaning of the target while delivering comparable results.^89,105^

The electrochemical ATR-SEIRAS experiments were performed in a customized cell. The CO_2_ reduction reaction occurs at the working electrode (WE) including Ag layer sputtered on top of the ATR crystal. A Pt counter electrode (CE), a Ag/AgCl reference electrode (RE), and a gas in- and outlet to purge CO_2_. The electrolyte used was KCl due to its invisibility for infrared radiation, making it suitable for these measurements to isolate the intermediate species formed during CO_2_ reduction on the catalyst surface. SEIRAS spectra were collected in a Brucker Vertex 70 modified FT-IR spectrometer, averaged over 72 scans at a resolution of 4 cm^–1^. These spectra were collected as reflectance of the signal and transformed into absorbance units (au) using the relation: *A* = −log(*R*/*R*_0_). The sample chamber accommodates the proprietary cell and an additional N_2_ purge (Figure S29).

Electrochemical routines were performed using a BioLogic SP-200 potentiostat. Before any spectroscopic measurement, the cell was purged for 30 min using 99.999% pure CO_2_ gas. This purge was also active during electrochemistry. Before starting SEIRAS experiments, the Ag thin film was activated by applying 6 cyclic voltammetries from +0.2 to −1.1 V vs Ag/AgCl. After this, background scans were collected at −0.5 V vs Ag/AgC, and consecutive scans every 50 mV during a linear sweep voltammetry at 2 mV/s. At −1.9 V vs Ag/AgCl, the potential was held for seven scans before being reversed to OCV at the same scan rate. The ATR-SEIRAS measurements were performed starting at the potential of −0.5 V vs Ag/AgCl and gradually increased to the potential of −1.9 V vs RHE. During the infrared measurements, the cell was connected to a potentiostat that supplied a fixed potential to the working electrode.

### H-Cell Electrochemical Measurements

Both glassy carbon and silver electrodes served as solid-based working electrodes individually for a systematic comparison. For each electrochemical reaction, the solution was saturated with either CO_2_ or Ar and the rest of the experiment was done in a sealed condition. All of the electrolysis was done under stirring conditions. The electrochemical studies were carried out using a CHI 660C potentiostat (CH Instruments, Austin, TX) with a three-electrode setup enclosed in a Faraday cage: glassy carbon (3 mm diameter) and sliver nanotubes (Ag) (working electrode), Pt wire (auxiliary), and Ag/AgCl (reference electrode). The whole reaction was conducted in 15 mL of 0.1 M KHCO_3_. The electrodes were connected to the cell via a Nafion membrane bridge. The CV measurements were applied with a positive initial scan polarity, 5 s quiet, and a scan rate of 0.1 V/s. All potentials were reported versus the Ag/AgCl reference electrode. Potentials were changed from Ag/AgCl (3 M KCl) to RHE (*E*_RHE_ = *E*_Ag/AgCl_ + 0.059 × pH + 0.210). In the neutral pH electrolyte, the current density will cause a local pH change near the electrode that makes the exact determination of the potential on an RHE challenging as a function of current density. Hence, at such low current densities, the change may be between 1 and 3 pH units.

The impedance measurements were from 0.1 Hz to 100 kHz frequency range with a 10 s quit time with a sampling rate of 4 points per decade, AC amplitude 10 mV, and bias potential 0.28 V. The impedance detection electrolyte was an aqueous solution containing 200 mM KNO_3_ and 2.5 mM K_3_[Fe(CN)_6_]/K_4_[Fe(CN)_6_] (1:1) as electroactive probes. The GC was equipped with a packed Molecular Sieve 5A capillary column and a packed HaySep D column. Helium (99.999%) was used as the carrier gas. A helium ionization detector (HID) was used to quantify H_2_ and CO concentrations.

### MEA Cell Electrochemical Measurements

All experiments were performed in a 5 cm^2^ area membrane electrode assembly (Dioxide materials) having a serpentine flow channel on both the anode and cathode endplates. A Sigracet 38 BC gas diffusion layer (GDL) of 6.25 cm^2^ area (2.5 cm × 2.5 cm) was used as the porous transport layer. A Ag catalyst layer was deposited on top of the microporous layer of GDL by direct current magnetron sputtering to obtain a thickness of 10 nm. Nickel foam (3 cm × 3 cm) was used as the anode. Ag GDE and Ni foam (Recemat BV) were combined with an oversized 16 cm^2^ (4 cm × 4 cm) Sustainion anion-exchange membrane (X37-50 Grade RT) to assemble the MEA. An exchange MEA configuration using 1 M KOH as the anolyte and humidified CO_2_ as a reactant at the cathode was fed into the reactor at a flow rate of 50 sccm.

The MEA was prepared by physical compression of the electrodes and endplates using a torque wrench, which were tightened to 4 Nm. This value was chosen to enhance the contact between the GDE and membrane while simultaneously ensuring that no physical damage occurred to the carbon GDE. A series of constant current electrolysis experiments were performed, and the gaseous products from the cell were analyzed using an online gas chromatography connected to the outlet of the cell equipped with two thermal conductivity detectors and a flame ionization detector. Constant current electrolysis from 10 to 200 mA/cm^2^ was performed for 1200 s at each current density. Aliquots were collected every 5 min during the reaction resulting in a total of four injections for each current density in 1200 s.

The flow rate at the outlet of the reactor was measured using a mass flow meter (Bronkhorst) to estimate the faradic efficiency of products accurately. A LABVIEW program was built and connected to the mass flow meter for continuous monitoring of the outlet flow rate. The outlet flow rate of the gas mixture (CO + H_2_ + residual CO_2_) from the reactor was measured (*V̇*_outlet_) using the mass flow meter, and the mole fractions of CO (*x*_CO_) and H_2_ (*x*_H_2__) were estimated from the GC injections.

### Flow Cell Electrochemical Measurements

A flow cell with three compartments composed of gas, catholyte, and anolyte chambers was used as reported from our group previously.^102^ CO_2_ was fed through a mass controller (Bronkhorst High-Tech BV) at a flow rate of 20 sccm. In all experiments, the catholyte (100 mL) and anolyte (100 mL) were 1 M KHCO_3_ (99.9% Sigma-Aldrich), supplied by a peristaltic pump at a rate of 20 mL/min. A Nafion 115 proton-exchange membrane was used to separate catholyte and anolyte. The electrochemically reacted gas and catholyte were sent into a gas-tight reservoir to balance the pressure at the gas and catholyte interfaces. Subsequently, gas was sent to GC for product analysis, while the catholyte circulated back to the catholyte chamber. The anolyte circulated through a different reservoir, which was open to the atmosphere to allow the anodic product O_2_ to escape. The pH of electrolytes was measured before and after each test using a pH meter (HANNA, HI-98191).

### Faradic Efficiency Calculation

To estimate the faradic efficiency of gaseous products, the mole fractions of CO and H_2_ were estimated from GC injections. The volume fraction of gas products from GC is equal to the mole fraction for ideal gases. The mole fraction of water vapor exiting the reactor was measured using a humidity sensor and was found to be 78% (*x*_H_2_O_ = 0.023). Since the sum of mole fractions is equal to 1, the mole fraction of CO_2_ exiting was calculated as eq 3
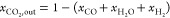
3

After calculating the mole fractions of all gaseous products, the volumetric flow rate at the outlet of the reactor was measured with the MFM and used to calculate the moles of each product
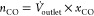
4
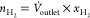
5
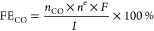
6Here, *n*_CO_ is the moles/s of CO produced, *n*^e^ is the number of electrons involved in CO_2_RR (2 for CO), *F* is 96,485 C/mol, and *I* is the applied current (in Amperes).

As shown previously,^47^ immobilization of the Ag onto GCE was achieved through a rapid and one-step electrodeposition technique (Figure S1). The Ag was prepared *in situ* on a clean GCE surface via electrodeposition of a 1 mM AgNO_3_ precursor solution with 0.1 M NaHCO_3_ under a constant applied potential of −0.2 V vs RHE for 200 s

The surface concentration (Γ) was calculated according to eq 7
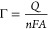
7where *Q* is the total charge (C), *n* is the number of electrons transferred, *F* is the Faraday constant (96,485 C/mol), and *A* is the electrode surface area (0.071 cm^2^).

The catalyst concentration of Ag-EPy-1, Ag-EPy-2, and Ag-EPy-3 was calculated as 4.5 × 10^–7^, 3.8 × 10^–7^, and 3.3 × 10^–7^ mol/cm^2^, respectively. These values are close to those in the case of glassy carbon electrodes: EPy-1 (4.7 × 10^–7^ mol/cm^2^), EPy-2 (3.9 × 10^–7^ mol/cm^2^), and EPy-3 (3.5 × 10^–7^ mol/cm^2^).

### Density Functional Theory (DFT) Calculations

DFT computations were performed using the Vienna Ab initio simulation package (VASP)^106^ and on Compute Canada clusters. In all computations, we used the projected augmented wave (PAW) pseudopotentials and the generalized gradient approximation (GGA) of Perdew–Burke–Ernzerhof (PBE) as their exchange–correlational functionals.^107^ A cutoff energy of 450 eV for the plane-wave basis sets and a 2 × 2 × 1 Γ-centered Monkhorst–Pack mesh for the k-point sampling in the first Brillouin zone, with a first-order Methfessel–Paxton smearing parameter σ of 0.1 eV, ensured that the energy convergence criteria are better than 1 meV for a vacuum of 20 Å or greater. The self-consistent field (SCF) convergence criterion is set to 1 × 10^–4^ eV for electronic iteration, and the ionic relaxation continued till the maximum force was less than 0.02 eV/Å that was updated by the conjugate gradient approach. Dipole corrections and spin polarization are implemented. The DFT-D3 method with the Becke–Jonson damping is performed for the van der Waals correction.

Reaction steps were simulated over the Ag(111) facet, as the most stable crystalline orientation of Ag, made of 100 silver atoms in four layers where the two top layers were allowed to be relaxed and the two bottom ones were fixed in their optimized position to represent the characteristics of the bulk silver atoms. Figures S21 and S22 include three different hybrid catalysts that were optimized after the addition of pyridines to the surface of the silver. To calculate the reaction energy diagram, the proton-coupled-electron-transfer (PCET) scheme^82^ was followed using the computational hydrogen electrode (CHE).^108^ *COOH and *CO demonstrate adsorbed carboxyl and carbon monoxide intermediates, respectively, where * denotes the catalyst surface. Optimized structures and equations used to calculate the reaction energy diagram are provided in the Supporting Information (eqs S1–S7). The electrostatic charge density around each ion is calculated by the Bader charge analysis method.^109^ VESTA software is used for the visualization.^110^

Reaction mechanism on the Ag 111 facet (* stands for the catalyst and i* is equivalent with adsorbed i)
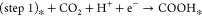

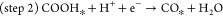


Therefore, for each step, writing the energy balance, we will have
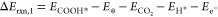

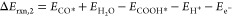


Modeling by proton-coupled electron transfer (PCET), we will have
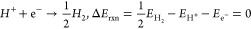


Thus, we can substitute (*E*_H^+^_ + *E*_e^–^_) by half of the *E*_H_2__, eventuating in
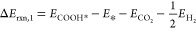

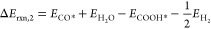


## Supporting Information Available

Reagents and chemicals, material characterizations, electrochemical measurements, 44 Figures, and 7 Tables (PDF)

## Notes

All authors have given approval to the final version of the manuscript.
