# Supplementary material for: CO2 Electrolysis via Surface-Engineering Electrografted Pyridines on Silver Catalysts
Source: ACS Catal. 2022 Jun 17;12(13):7862–76. doi: 10.1021/acscatal.2c01654 (PMC9251727; doi:10.1021/acscatal.2c01654)
Supplement: Supplementary file 1 — cs2c01654_si_001.pdf [file cs2c01654_si_001.pdf]

Supporting Information

CO_2 Electrolysis via Surface-Engineering Electrografted Pyridines on Silver Catalysts

Maryam Abdinejad,[a] Erdem Irtem,[a] Amirhossein Farzi,[b] Mark Sassenburg,[a] Siddhartha Subramanian,[a] Hugo-Pieter Iglesias van Montfort,[a] Davide Ripepi,[a] Mengran Li,[a] Joost Middelkoop,[a] Ali Seifitokaldani*[b] and Thomas Burdyny*[a]

a) Department of Chemical Engineering, Delft University of Technology, Van der Maasweg 9

2629 HZ Delft, the Netherlands

b) Department of Chemical Engineering, McGill University, Montreal H3A 0C5, Canada

T.E.Burdyny@tudelft.nl and ali.seifitokaldani@mcgill.

Number of Pages: 36
Number of Figures: 44
Number of tables: 7

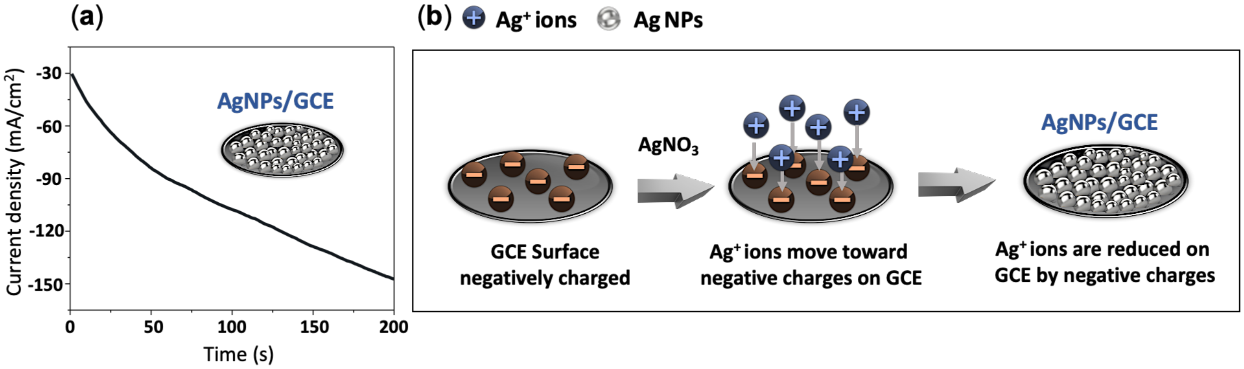

Figure S1. a) *In situ* synthesis of silver nanoparticles onto glassy carbon surface using $AgNO_3$ (1 mM) and $NaHCO_3$ (0.1 M) solution under constant potential of -0.2 V *vs* Ag/AgCl for 200 s; b) Schematic of *in situ* electrodeposition of silver nanoparticles on glassy carbon electrode.

Figure S2c shows EPy-3 (3625 ± 46.1 Ω) with the greatest charge transfer resistance (R_{ct}) followed by EPy-1 (2806.2 ± 42.5 Ω), EPy-2 (1893 ± 41.3 Ω) and bare glassy carbon electrode (134.1 ± 2.18 Ω), respectively.

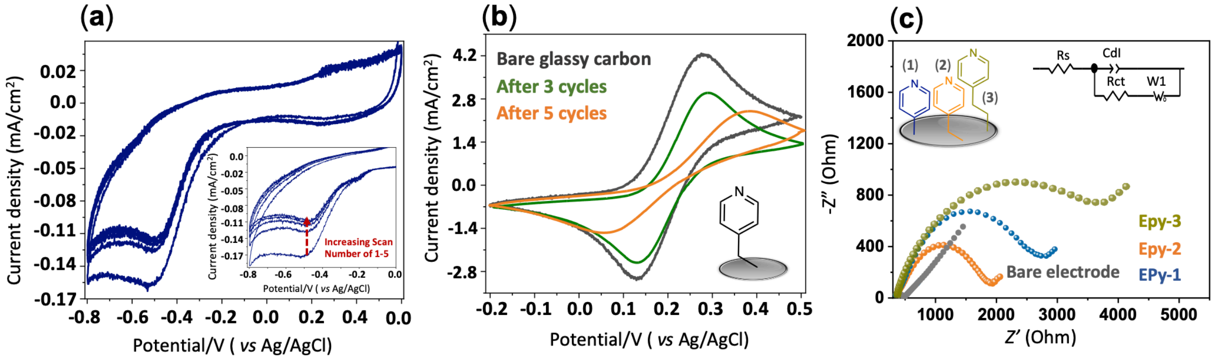

Figure S2. (a) Electrografting voltammogram of 5 mM Py-2 onto glassy carbon electrode in 2 mM $NaNO_2$ and 0.5 M HCl at a scan rate of 50 mV/s; (b) CV of the ferrocyanide redox probe before and after 3-5 cycles electrografting of Py-2 onto glassy carbon electrode at a scan rate of 50 mV/s; (c) Nyquist diagrams of bare glassy carbon, and EPy-x in 2.5 mM $[Fe(CN)_6]^{3-/4-}$ and 200 mM KNO_3.

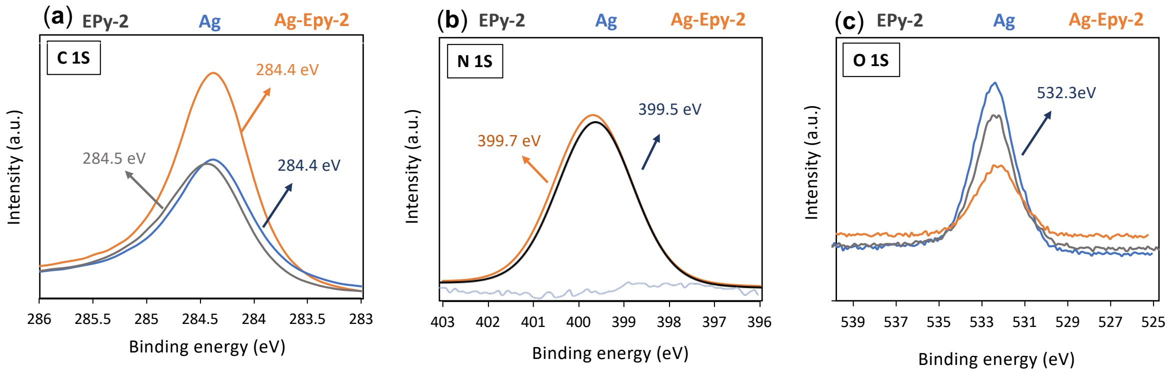

Figure S3. X-ray photoelectron spectroscopy characterization of modified silver electrode: a) C 1S; b) N 1S; and c) O 1s spectra of Ag, Ag-EPy-2, and EPy-2.

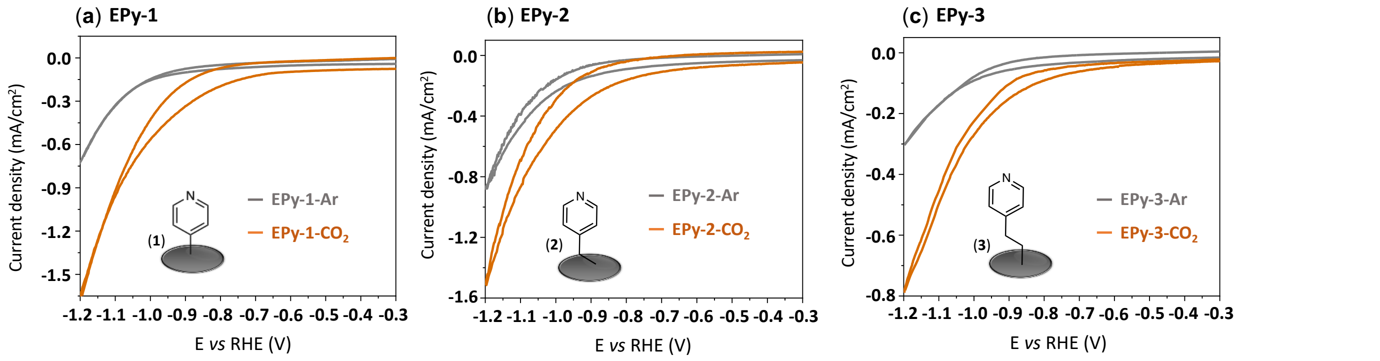

Figure S4. Cyclic voltammetry (CV) of heterogeneous electrografted pyridine catalysts onto glassy carbon electrode (GCE): a) EPy-1; b) EPy-2; and c) EPy-3 under Ar and CO_2 in 0.1 M $KHCO_3$.

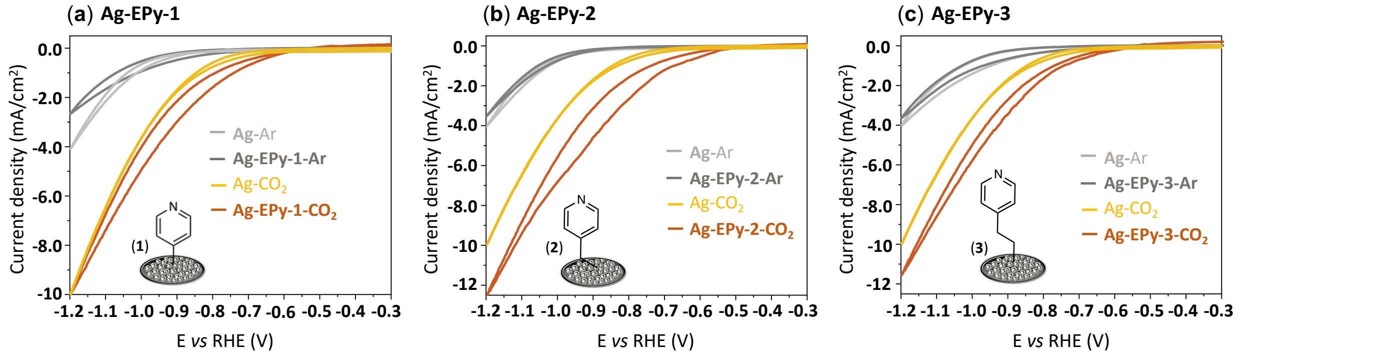

Figure S5. Cyclic voltammetry (CV) of heterogeneous electrografted pyridine electrocatalysts onto silver electrode: a) Ag-EPy-1; b) Ag-EPy-2; and c) Ag-EPy-3 under Ar and CO_2 in 0.1 M $KHCO_3$.

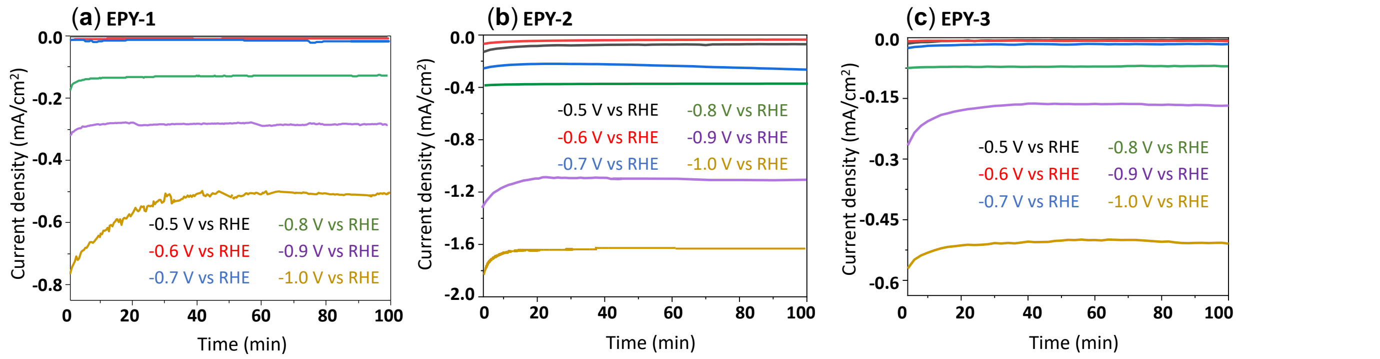

Figure S6. Chronoamperometry comparison of heterogeneous a) EPy-1; b) EPy-2; and c) EPy-3 electrocatalysts under CO_2 at -0.5, -0.6, -0.7, -0.8, -0.9, and -1.0 V *vs* RHE in 0.1 M $KHCO_3$.

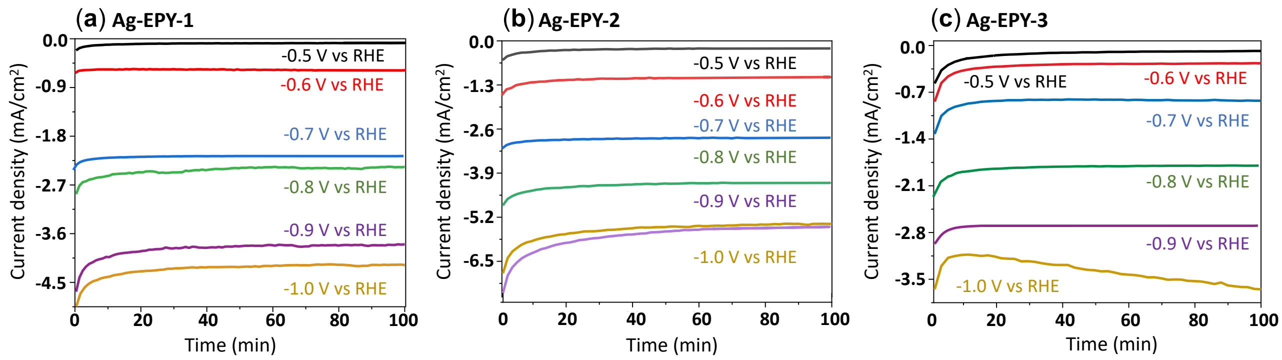

Figure S7. Chronoamperometry comparison of heterogeneous a) Ag-EPy-1; b) Ag-EPy-2; and c) Ag-EPy-3 electrocatalysts under CO_2 at -0.5, -0.6, -0.7, -0.8, -0.9, and -1.0 V *vs* RHE in 0.1 M $KHCO_3$.

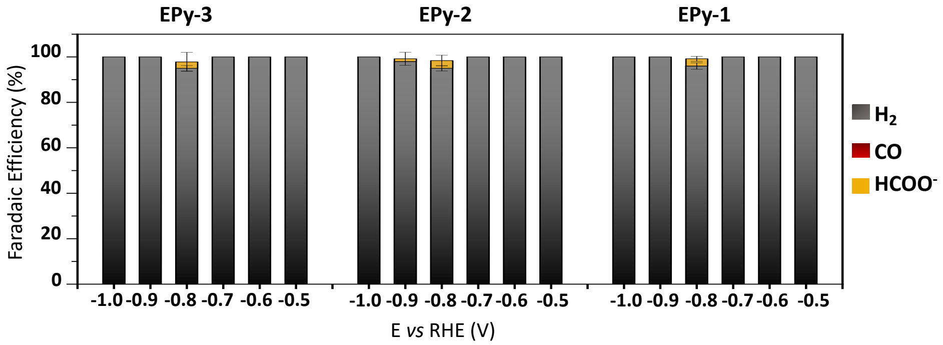

Figure S8. Faradaic efficiency (FE) of heterogeneous EPys electrocatalysts under CO_2 at -0.5, -0.6, -0.7, -0.8, -0.9, and -1.0 V *vs* RHE in 0.1 M $KHCO_3$.

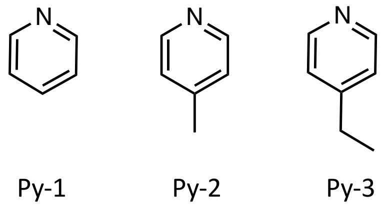

Figure S9. Molecular structure of homogeneous pyridine catalysts Py-1, Py-2, and Py-3

The homogeneous electrocatalytic activity of compounds Py-x (5 mM) was evaluated at both GCE and Ag working electrodes in 0.1 M $KHCO_3$ after saturation first with Ar, then with CO_2 (Figure S10 and S11). Under CO_2-saturation, a distinct one-electron reduction wave was observed, which is attributed to the catalytic current of CO_2 electroreduction and/or HER.

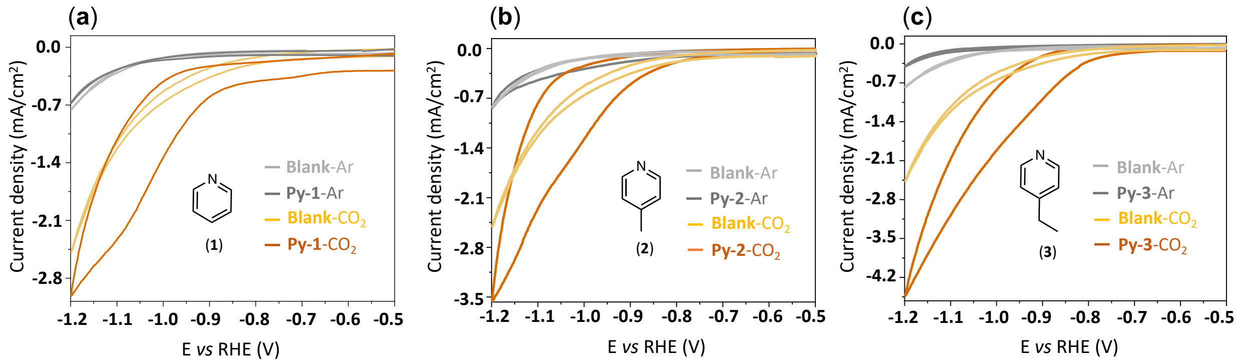

Figure S10. Cyclic voltammetry (CV) of a solution of homogeneous 5 mM (a) Py-1; (b) Py-2; and (c) Py-3 under Ar and CO_2 in 0.1 M $KHCO_3$.

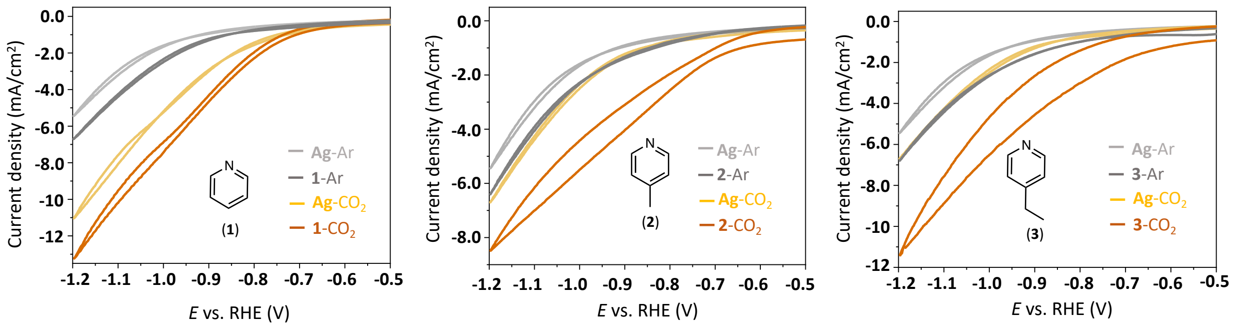

Figure S11. Cyclic voltammetry (CV) of a solution of homogeneous 5 mM (a) Ag-Py-1; (b) Ag-Py-2; and (c) Ag-Py-3 under Ar and CO_2 in 0.1 M $KHCO_3$.

Comparison of the CV of Py-x (Figure S12a) and Ag-Py-x (Figure S12b) shows the catalytic activity of Py-3 is slightly greater than that of Py-1 and Py-2. The same trend was observed in the case of using silver electrode (Ag-Py-x) confirming the integral role of extra carbon chain in improving the catalytic efficiency of the pyridine species. Exposure of the catalysts to CO_2 resulted a dramatic increase to the current density beginning at ~-0.8 V *vs* RHE with glassy carbon electrode. Replacing the glassy carbon working electrode with silver, a higher current density experienced with a noticeable negative shift at lower overpotential energy (~-0.7 V *vs* RHE), which could be due to the activity of Ag towards CO_2RR.

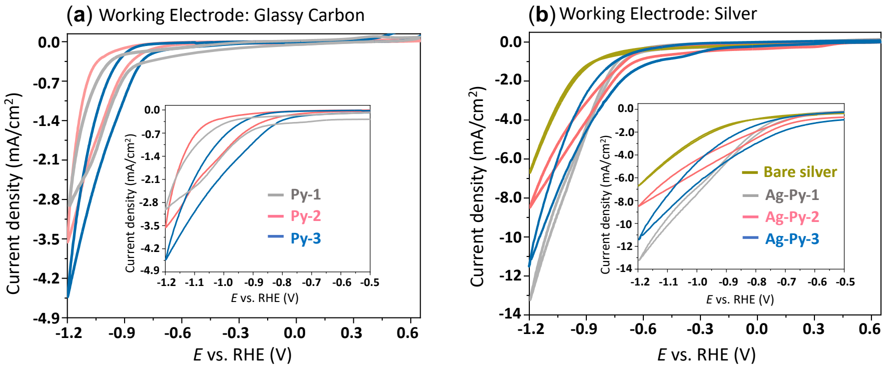

Figure S12. Cyclic voltammetry (CV) comparison of homogeneous pyridines at (a) glassy carbon electrode (Py-1, Py-2, and Py-3); and (b) silver electrode Ag-Py-1, Ag-Py-2 and Ag-Py-3 under CO_2 in 0.1 M $KHCO_3$.

Direct comparison of the best homogeneous catalyst (Py-3) at GCE, confirms upon saturation of the solution with CO_2, higher catalytic activity is obtained at Ag at lower overpotential confirming the better catalytic performance arise from both Ag and pyridine group (Figure 13a). A closer look at the CVs in Figure S13b, shows an irreversible reduction peak at –0.51 V *vs* RHE at GCE assigned to PyH^+ reduction to pyridine.[1] This peak shifted to more positive potential of -0.34 V using Ag, which is likely is a result of the increasing of the PyH^+ in the electrode interface as well as interaction between PyH^+ species and silver electrode surface (Figure S13c). The achieved results are in agreement with the previous studies.[1,2]

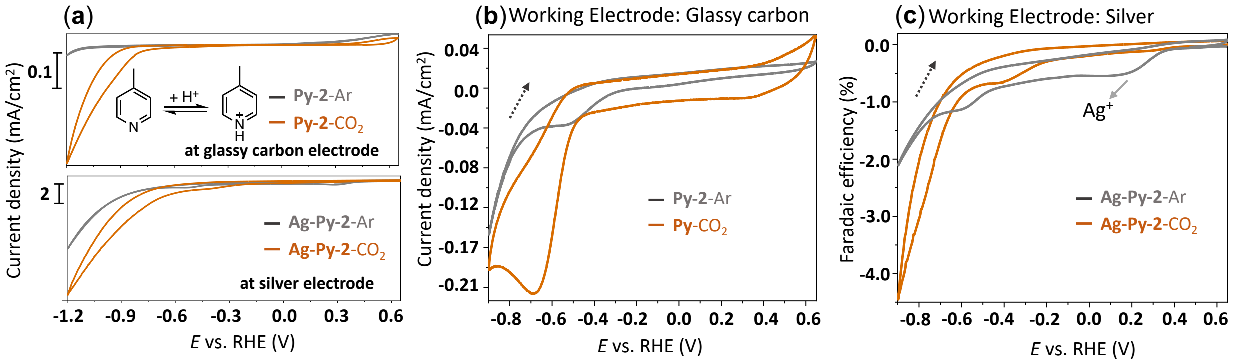

Figure S13. (a) CV comparison of the homogeneous pyridine catalyst at glassy carbon (top) and silver (bottom) working electrodes, under Ar and CO_2 in 0.1 M $KHCO_3$. Cyclic voltammetry (CV) comparison of a homogeneous solution of Py-2 (5 mM); and (c) silver electrodes under Ar and CO_2 in 0.1 M $KHCO_3$.

To determine the optimal potential for electrochemical CO_2 reduction, chronoamperometry studies of Py-x (Figure S14) and Ag-Py-x (Figure S15) were performed at -0.5, -0.6, -0.7, -0.8, -0.9 and -1.0 V *vs* RHE. Using the GCE working electrode, after purging the electrolyte with CO_2, H_2 was

observed as a main product with a trace amount of formate (2-3.5%) -0.5 to -0.8 V *vs* RHE. Therefore, the increase in current after purging the electrolytes with CO_2 can be attributed to CO_2RR current, and RHE which mainly belongs to the reduction of PyH^+ and carbonic acid in the electrolyte solutions.[3]

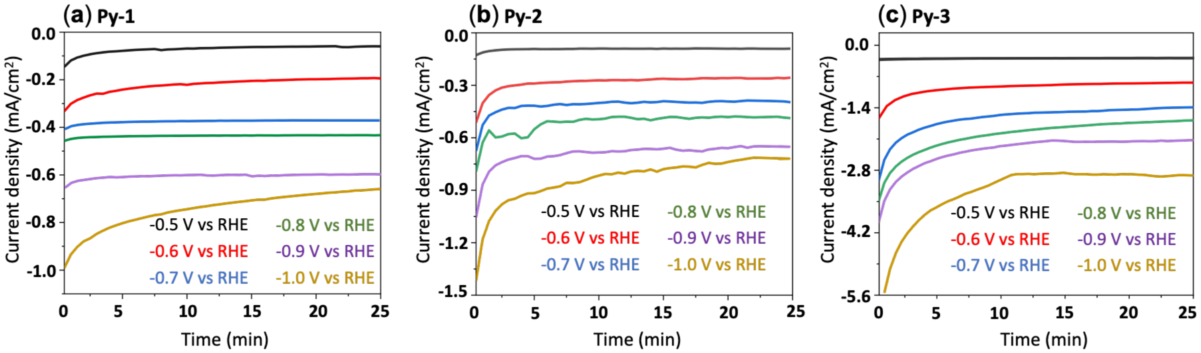

Figure S14. Chronoamperometry comparison of homogeneous a) Py-1; b) Py-2; and c) Py-3 electrocatalysts under CO_2 at -0.5, -0.6, -0.7, -0.8, -0.9, and -1.0 V *vs* RHE in 0.1 M $KHCO_3$ at glassy carbon working electrode.

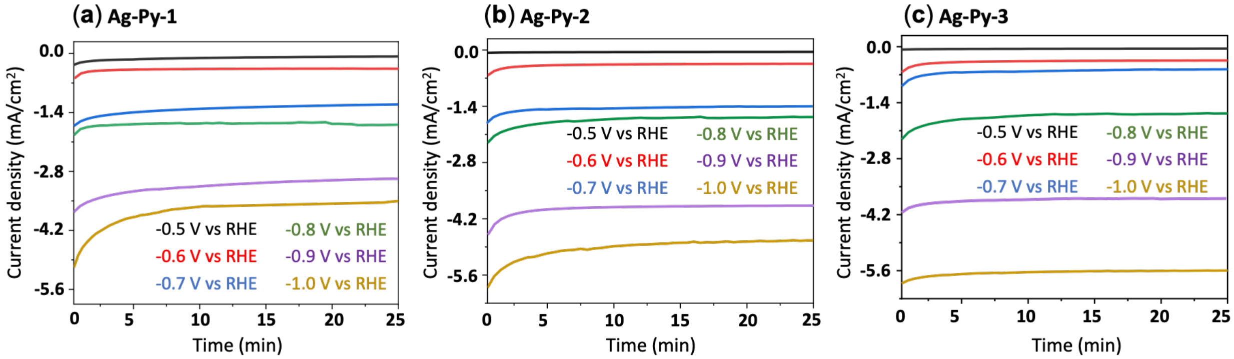

Figure S15. Chronoamperometry comparison of homogeneous a) Ag-Py-1; b) Ag-Py-2; and c) Ag-Py-3 electrocatalysts under CO_2 at -0.5, -0.6, -0.7, -0.8, -0.9, and -1.0 V *vs* RHE in 0.1 M $KHCO_3$ at silver working electrode.

Using the Ag working electrode, H_2 and CO were achieved as the primary products and no liquid products were observed within our detection limits. Among the catalysts performed in Figure S16 and S17, Ag-Py-3 demonstrated the highest catalytic selectivity with FE_{CO}: 41%; and *j*: 1.7 mA/cm^2 at -0.8 V *vs* RHE, which is slightly higher than bare Ag (FE_{CO}: 36%, *j*: 1.1 mA/cm^2) at the same potential. There were no noticeable differences between catalyst 1 and 2 (FE_{CO}: ~38%, j: 1.7 mA/cm^2) at the same overpotential. The higher selectivity achieved by catalyst Py-3 could be due to having longer chain. This observation of an earlier onset potential for Ag compared to glassy carbon electrode is also in agreement with the better performance of the sliver electrode catalyst.

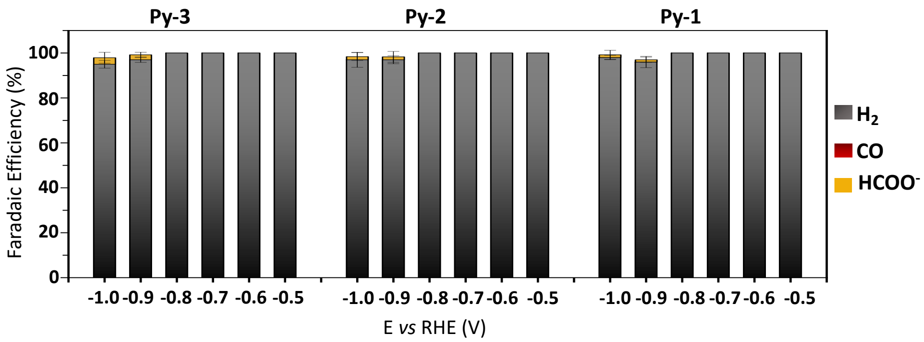

Figure S16. Faradaic efficiency (FE) of homogeneous a) Py-1; b) Py-2; and c) Py-3 electrocatalysts at glassy carbon working electrode under CO_2 at -0.5, -0.6, -0.7, -0.8, -0.9, and -1.0 V *vs* RHE in 0.1 M $KHCO_3$.

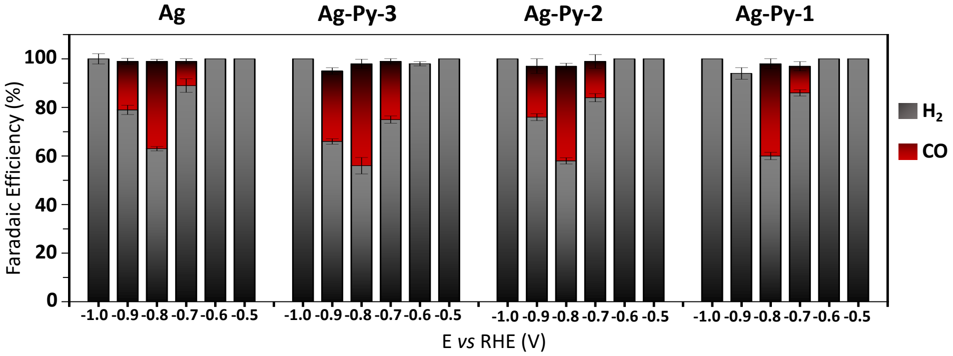

Figure S17. Faradaic efficiency (FE) of homogeneous a) Ag-Py-1; b) Ag-Py-2; and c) Ag-Py-3 at silver electrode under CO_2 at -0.5, -0.6, -0.7, -0.8, -0.9, and -1.0 V *vs* RHE in 0.1 M $KHCO_3$.

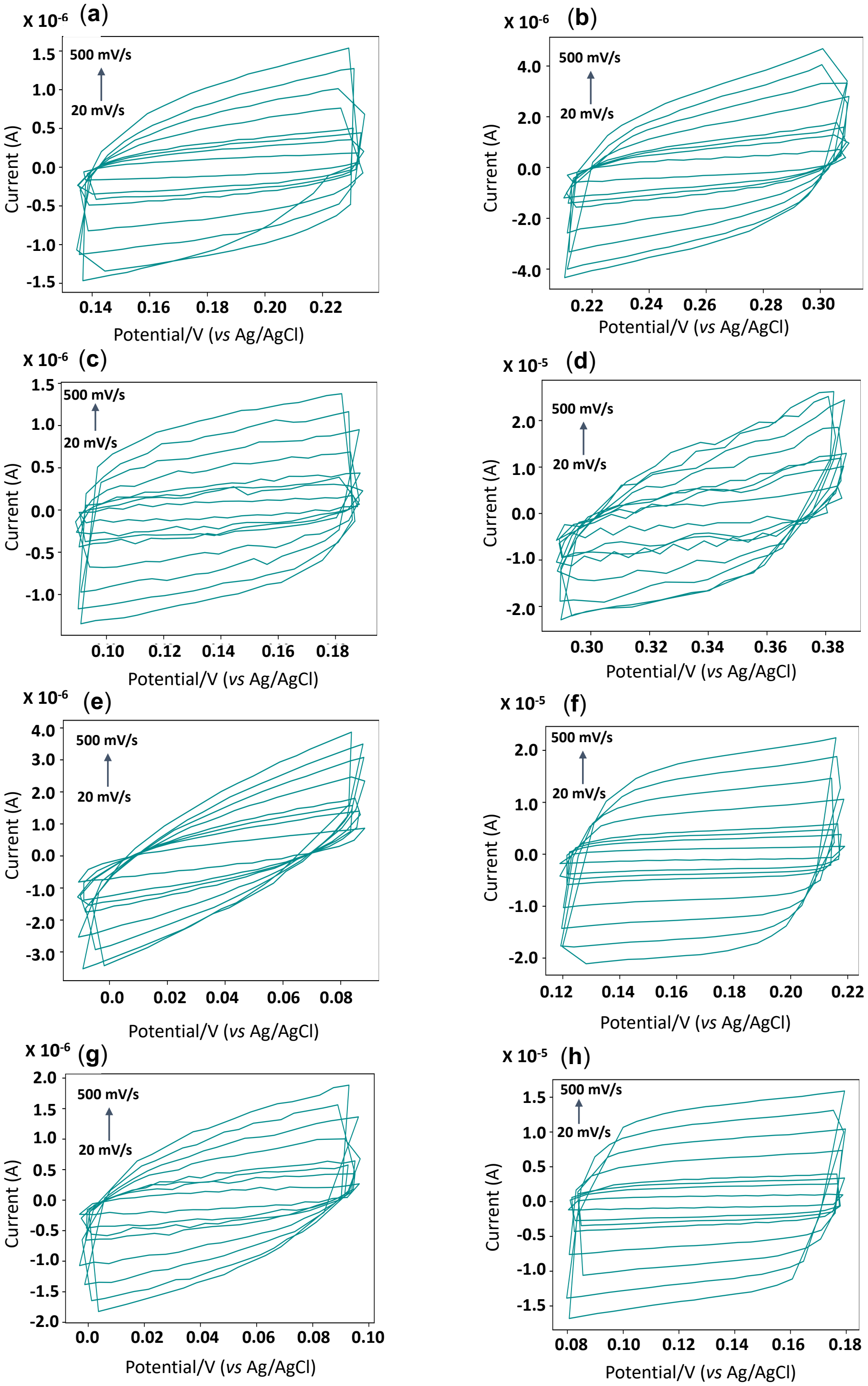

Figure S18. Cyclic voltammetry (CV) of (a) bare glassy carbon electrode; (b) bare Ag electrode; (c) EPy-1; (d) Ag-EPy-1; (e) EPy-2; (f) Ag-EPy-2; (g) EPy-3; and (h) Ag-EPy-3 at the scan rate of 100, 120, 160, 180 and 200 mV/s in 2.5 mM $[Fe(CN)_6]^{3-/4-}$ and 0.2 M KNO_3.

The electrochemically active surface area (ECSA) of compound 2 compared to 1 and 3 was calculated before and after electrografting with the pyridine complexes through Eq. 1:[4,5]

$$\text{Eq. 1: } A = \text{slope}/(268600 \times n^{3/2} \times D^{1/2} \times c)$$

Where n is the number of electrons transferred in the redox reaction (n = 1), D is the diffusion coefficient of ferrocene probe (7×10^{-6} cm^2/s) and c is the concentration of ferrocene (2.5×10^{-3} mol/cm^3).

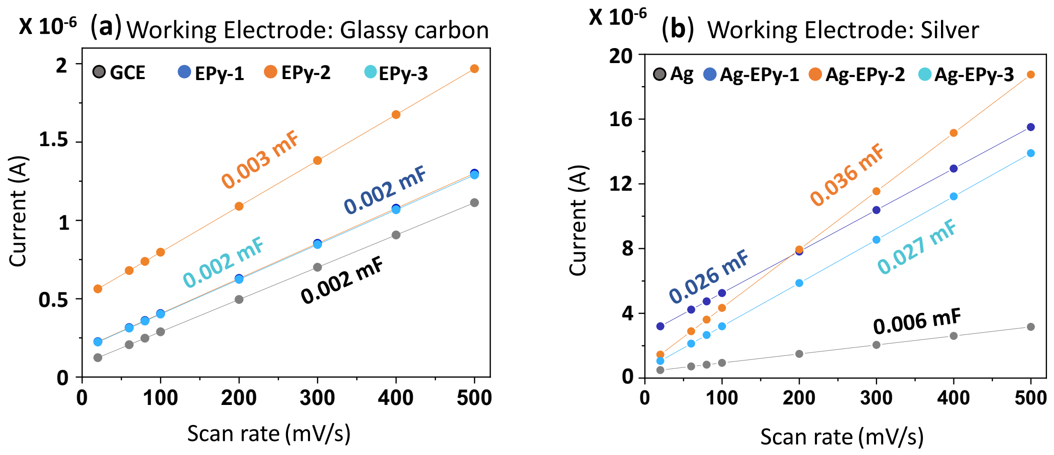

Figure S19. Plot of forward peak current density *vs* the square root of: (a) EPy-x at glassy carbon; and (b) Ag-EPy-x at silver electrode with the scan rates of 20, 60, 80, 100, 200, 300, 400, and 500 mV/s in 0.1M $KHCO_3$.

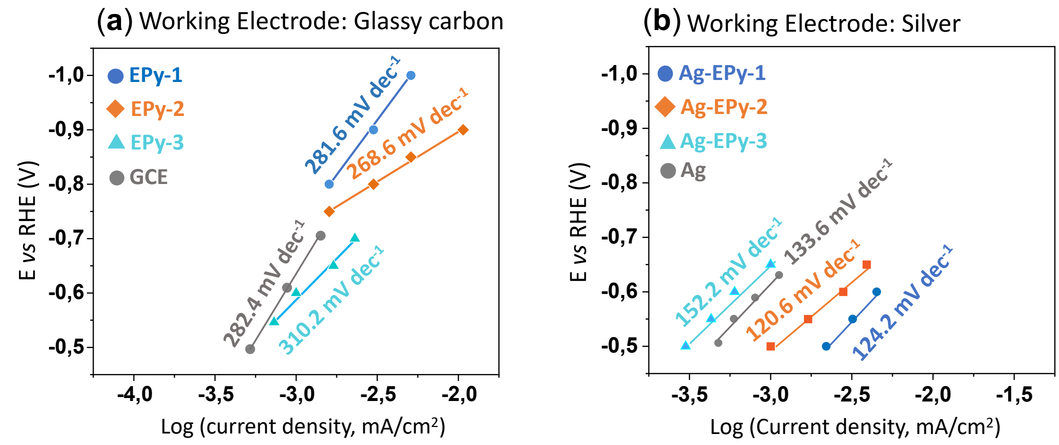

Figure S20. Tafel slopes for the current density of (a) EPy-x; and (b) Ag-EPy-x at -0.5, -0.6, -0.7, -0.8, -0.9, and -1 V *vs* RHE in 0.1 M $KHCO_3$.

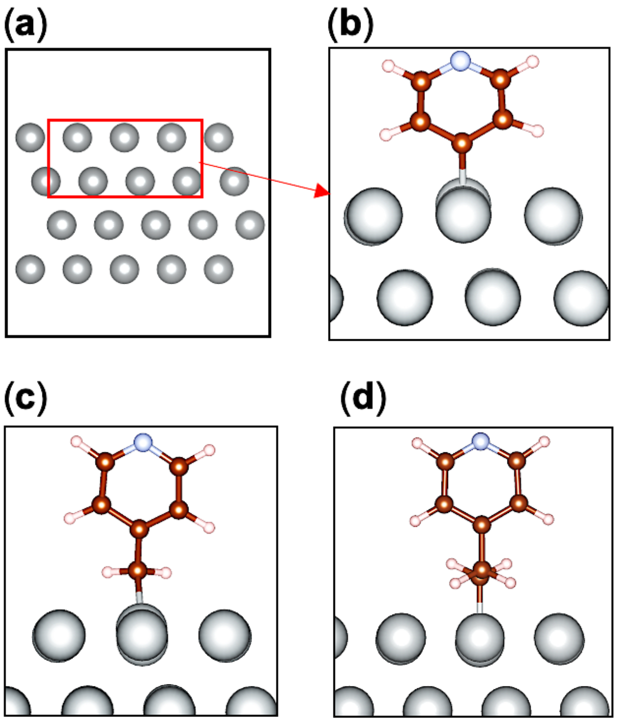

Figure S21. 111 slabs of (a) bare Ag, (b) Ag-EPy-1, (c) Ag-EPy-2 and (d) Ag-EPy-3 from side view.

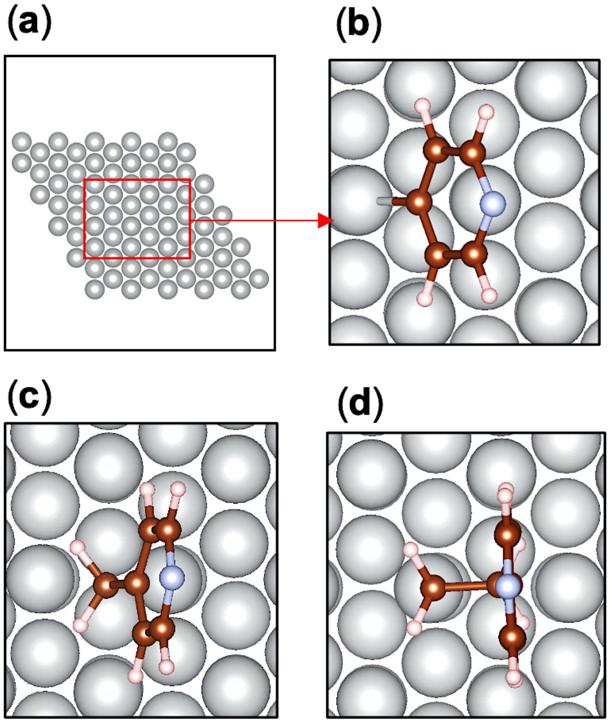

Figure S22. 111 slabs of (a) bare Ag, (b) Ag-EPy-1, (c) Ag-EPy-2, and (d) Ag-EPy-3 from top view.

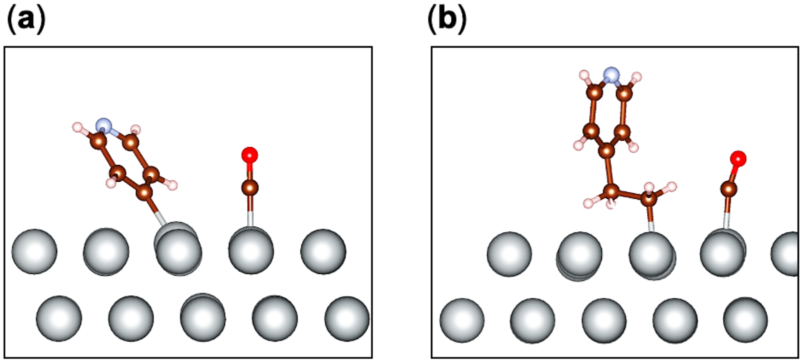

Figure S23. *CO adsorbed on 111 facets of (a) Ag-EPy-1, and (b) Ag-EPy-3.

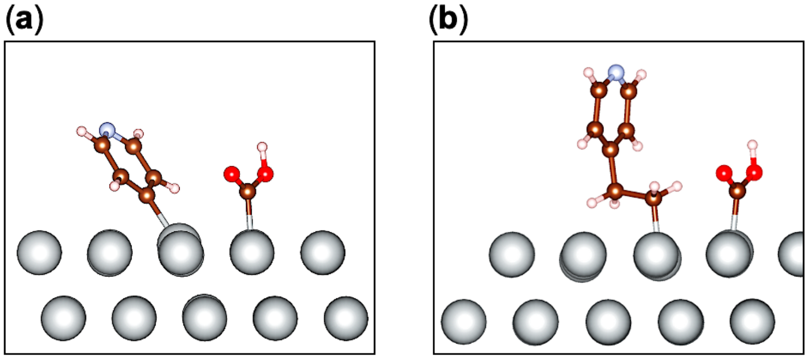

Figure S24. *COOH adsorbed on 111 facets of (a) Ag-EPy-1, (b) Ag-EPy-3.

DFT energies and calculated reaction energies are tabulated in Table S1 to S3. As depicted in Figure 4a the superior performance of Ag-EPy-1 is clearly shown in the output plots, suggesting a decrease in the activation barrier of the formation of the reaction's key intermediate (COOH*) by adding these two pyridine species to the surface of silver. These results also show the lower activity of Ag-EPy-3 in comparison with other two pyridine species.

Table S1 – Energies of optimized structures in eV

Catalyst	Bare Ag	Ag-EPy-1	Ag-EPy-2	Ag-EPy-3
**Energy (eV)**	-255.067	-320.715	-337.222	-353.298
**Catalyst + Surface species**	Ag + COOH	Ag-EPy-1 + COOH	Ag-EPy-2 + COOH	Ag-EPy-3 + COOH
**Energy (eV)**	-280.616	-346.287	-362.798	-378.824
**Catalyst + Surface species**	Ag + CO	Ag-EPy-1 + CO	Ag-EPy-2 + CO	Ag-EPy-3 + CO
**Energy (eV)**	-270.011	-335.724	-352.180	-368.260

Catalyst	Bare Ag	Ag-EPy-1	Ag-EPy-2	Ag-EPy-3
**Energy (eV)**	-255.067	-320.715	-337.222	-353.298
**Catalyst + Surface species**	Ag + COOH	Ag-EPy-1 + COOH	Ag-EPy-2 + COOH	Ag-EPy-3 + COOH
**Energy (eV)**	-280.616	-346.287	-362.798	-378.824
**Catalyst + Surface species**	Ag + CO	Ag-EPy-1 + CO	Ag-EPy-2 + CO	Ag-EPy-3 + CO
**Energy (eV)**	-270.011	-335.724	-352.180	-368.260

Table S2 – Energies of isolated molecules in eV

Species	CO_2	H_2O	H_2	CO
Energy (eV)	-22.973	-14.216	-6.763	-14.788

Table S3 – Reaction step energies in eV

step \ Catalyst	Bare Ag	Ag-EPy-1	Ag-EPy-2	Ag-EPy-3
step 1	0.804	0.783	0.779	0.828
step 2	-0.230	-0.242	-0.217	-0.262

Qualitative enhancement in charge distribution of the surface

To investigate the effect of adding adsorbents to the surface, charge distributions of isolated pyridine molecules and Ag surface have been subtracted from that of the electrografted Ag-EPy-x species. The results have been depicted in Figure 4b, and Figure S25-26. These Figures show how the addition of electrografted pyridines changes the uniformity of the charge distribution on the surface of silver. They also show the difference between the effect of three pyridine species on the surface. In Figure S26, the heterocyclic effect is almost negligible in the Ag-EPy-3 which may attribute to the larger distance between the pyridine ring and the surface, caused by the longer chain. In this case, we see almost no electronegative activity from the nitrogen.

The other important observation is the linear adsorption of *COOH over the silver atoms in all three cases. They also exhibit the affected adsorption sites that have the most significant charge donation (yellow clouds). To analyze how different the charge donation is from one catalyst to the other, we looked into the main three adsorbing Ag atoms (tagged by Ag1, Ag2 and Ag3) and investigated their corresponding charge donation. To quantify the analysis, the Bader charge analysis is performed which provides the oxidation states of all atoms, before and after the adsorption of *COOH intermediate.

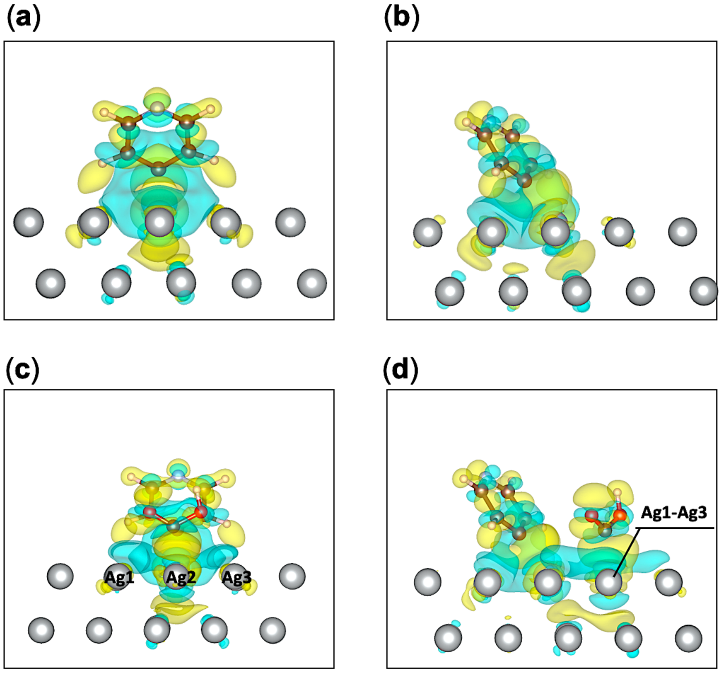

Figure S25 – Deviations in charge densities after electrografting Ag-EPy-1 from front (a) and side (b) views and after the adsorption of *COOH from (c) front, (d) side views.

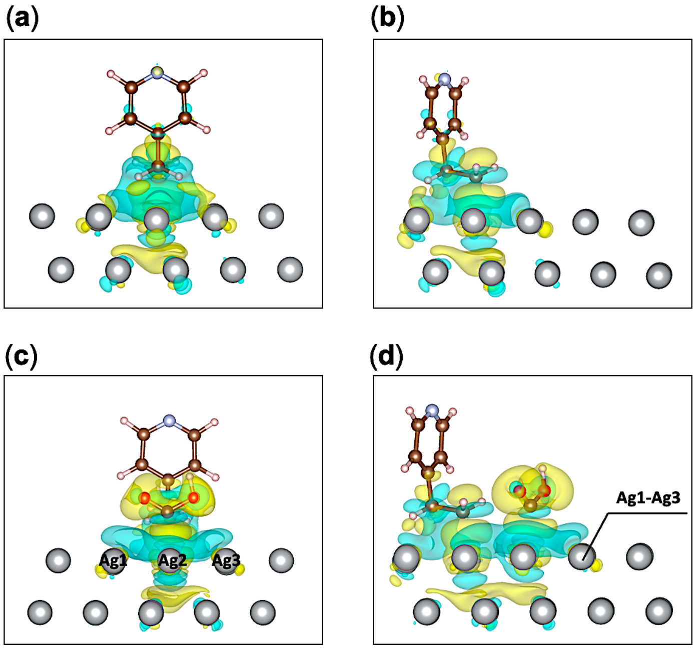

Figure S26 – Deviations in charge densities after electrografting Ag-EPy-3 from front (a) and side (b) views and after the adsorption of *COOH from (c) front, and (d) side views.

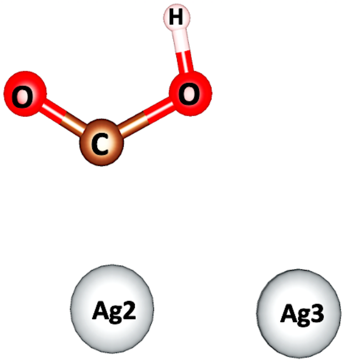

Figure S27 – The adsorption sites tabulated in table S4.

Table S4 – Oxidation states and charge donated by Ag atoms during the COOH adsorption for all Ag-pyridine species. Ag1, Ag2 and Ag3 corresponding to adsorption sites shown in Figure 4b and Figure S25-S26.

Case	Species	Ag1	Ag2	Ag3	Average charge donation (electrons/adsorbed COOH)
**EPy-1**	Ag-EPy-1	- 0.0243	- 0.0163	- 0.0187	-
	Ag-EPy-1 + COOH	0.0353	0.059	0.0219	-
	Electron donation	0.0596	0.0753	0.0406	0.1755
**EPy-2**	Ag-EPy-2	- 0.0277	- 0.0106	- 0.0314	-
	Ag-EPy-2 + COOH	0.0239	0.0692	0.0155	-
	Electron donation	0.0516	0.0798	0.0469	0.1783
**EPy-3**	Ag-EPy-3	- 0.0199	0.0138	0.0089	-
	Ag-EPy-3 + COOH	0.0451	0.0886	0.0362	-
	Electron donation	0.065	0.0748	0.0273	0.1671

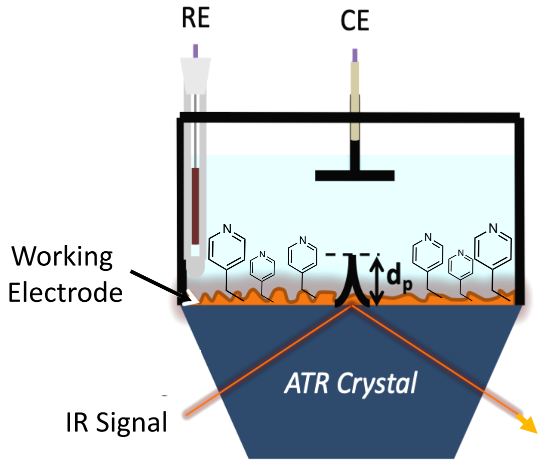

Figure S28. Proprietary cell set up used for enhanced infrared absorption spectroscopy (SEIRAS) measurement.

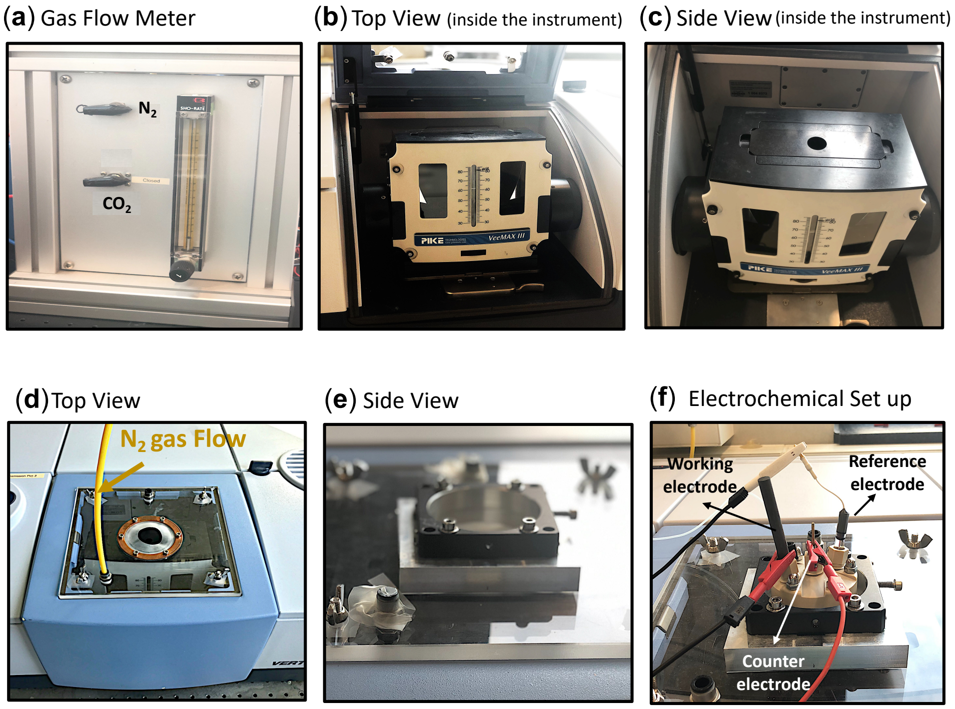

Figure S29. Modified sample compartment of the Brucker Vertex 70 to accommodate the H-cell. The silicone diaphragm wraps around the cell and seals the compartment, which is purged by an additional N_2 inlet (yellow) to over-pressurize the compartment and avoid air contamination.

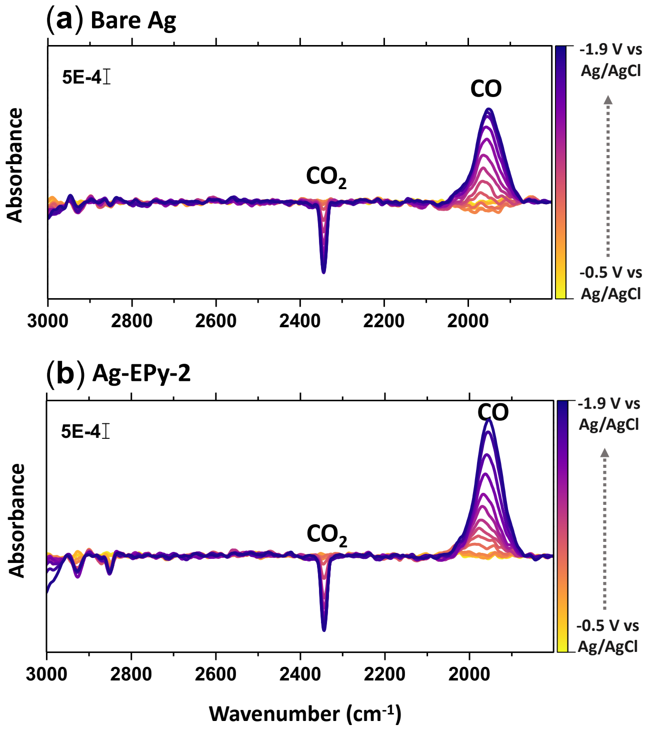

Figure S30. ATR-SEIRA spectra of (a) Ag; and (b) Ag-EPy-2 at 0.1 M KCl under CO_2

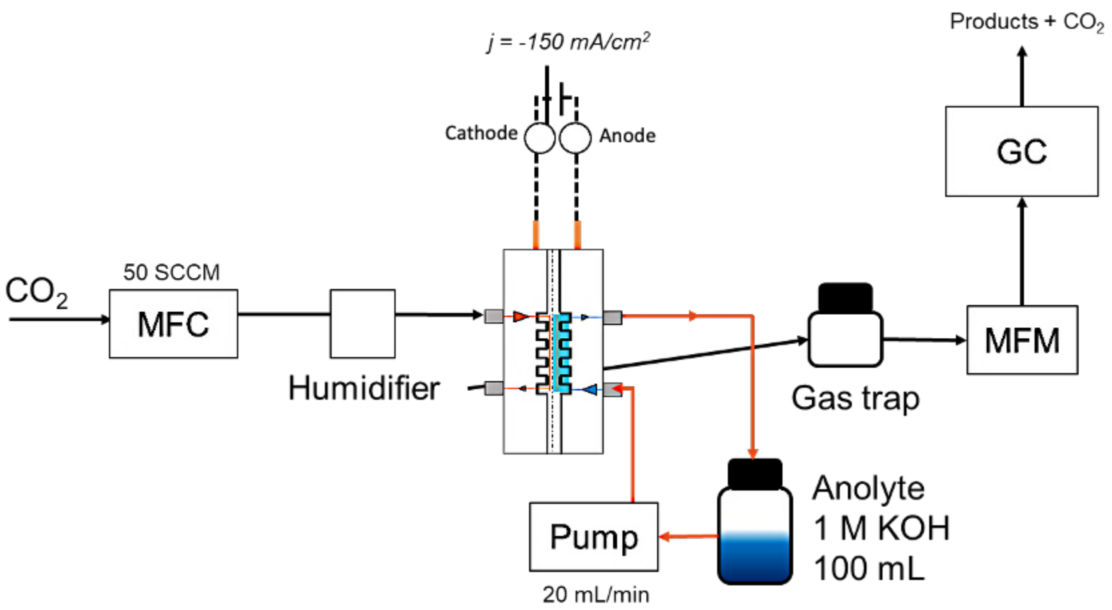

Figure S31. Schematic of MEA-type reactor

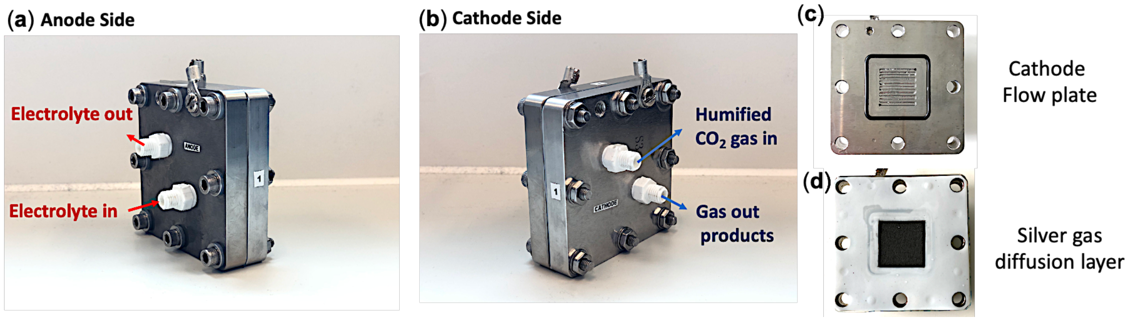

Figure S32. MEA cell set up for the electrochemical reduction of CO_2

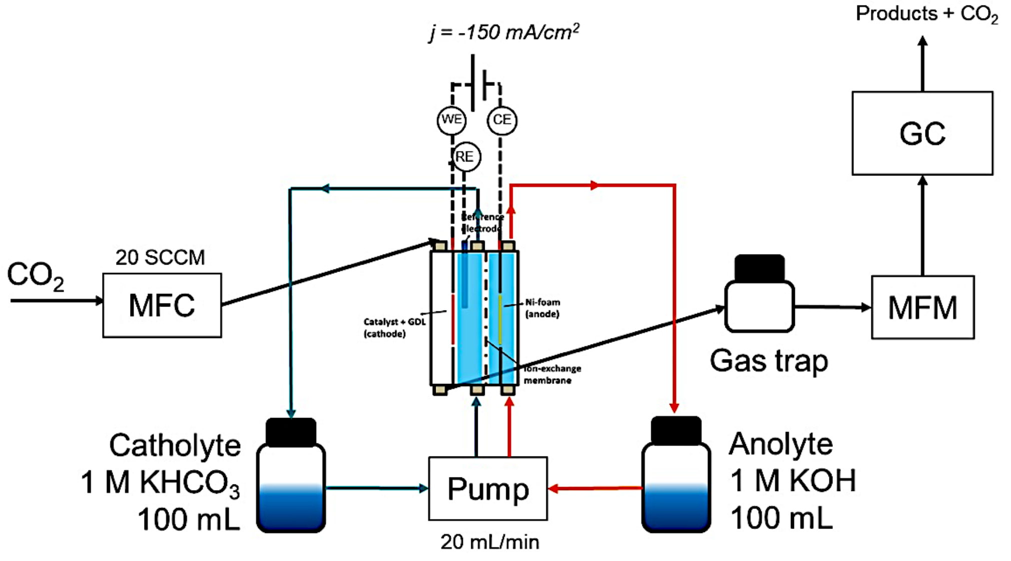

Figure S33. Schematic of the flow cell reactor

Electroreduction of CO_2 in the gas phase flow cell was performed at current densities of 10, 25, 50, 100, and 150 mA/cm^2 in 1 M $KHNO_3$ for both EPy-2 and bare Ag-EPy-2 for a systematic comparison (Figure S34, Table S7). CO and formate products were observed at all applied potentials. Using bare Ag electrode, the highest FE_{CO} was observed initially at 10 and 25 mA/cm^2; however, as the current density increased, CO production decreased substantially from 75% at 10 mA/cm^2 to 30% at 150 mA/cm^2 (Figure 5b). The reverse trend was observed using the electrografted Ag-EPy-2. With the influence of the pyridine layer, H_2 production was successfully suppressed from 55% in Ag to 33% in Ag-EPy-2 at 150 mA/cm^2. Overall, the amount of CO produced was improved from 30% in Ag to 58% in Ag-EPy-2 at the same current density. Considering the trade-off of increased current densities with the drawbacks of lower selectivity and electrode stability, 150 mA/cm^2 was determined to be the best for CO_2RR in the case of Ag-EPy-2.

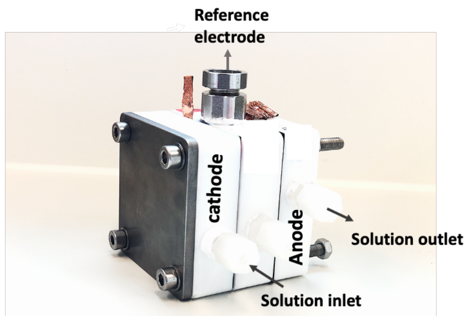

Figure S34. GDE-flow cell type electrolyzer for the electrochemical reduction of CO_2 using a liquid phase flow cell.

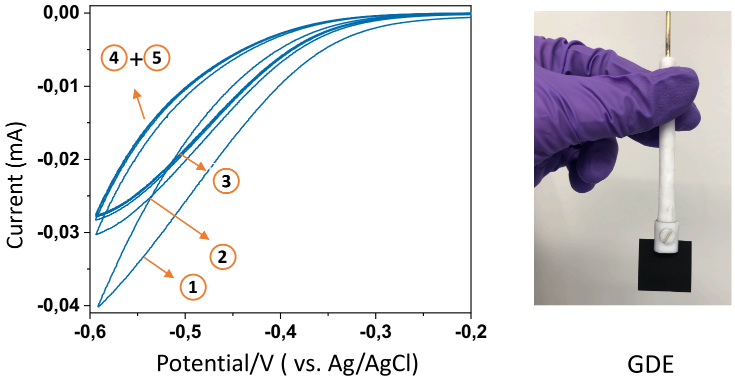

Figure S35. Electrografting voltammogram of 5 mM compound Ag-EPy-2 onto gas diffusion electrode (GDE) in 2 mM $NaNO_2$ and 0.5 M HCl at a scan rate of 50 mV/s

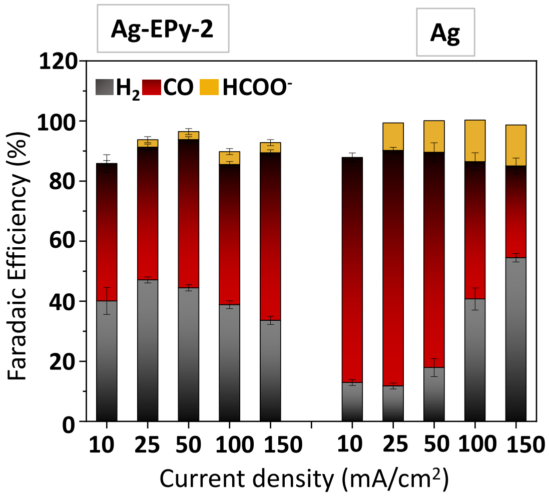

Figure S36. Faradaic efficiency (FE) comparison of the bare Ag and Ag-EPy-2 at current densities of 10, 25, 50, 100, and 150 mA/cm^2 in 1 M $KHCO_3$ using a liquid phase flow cell.

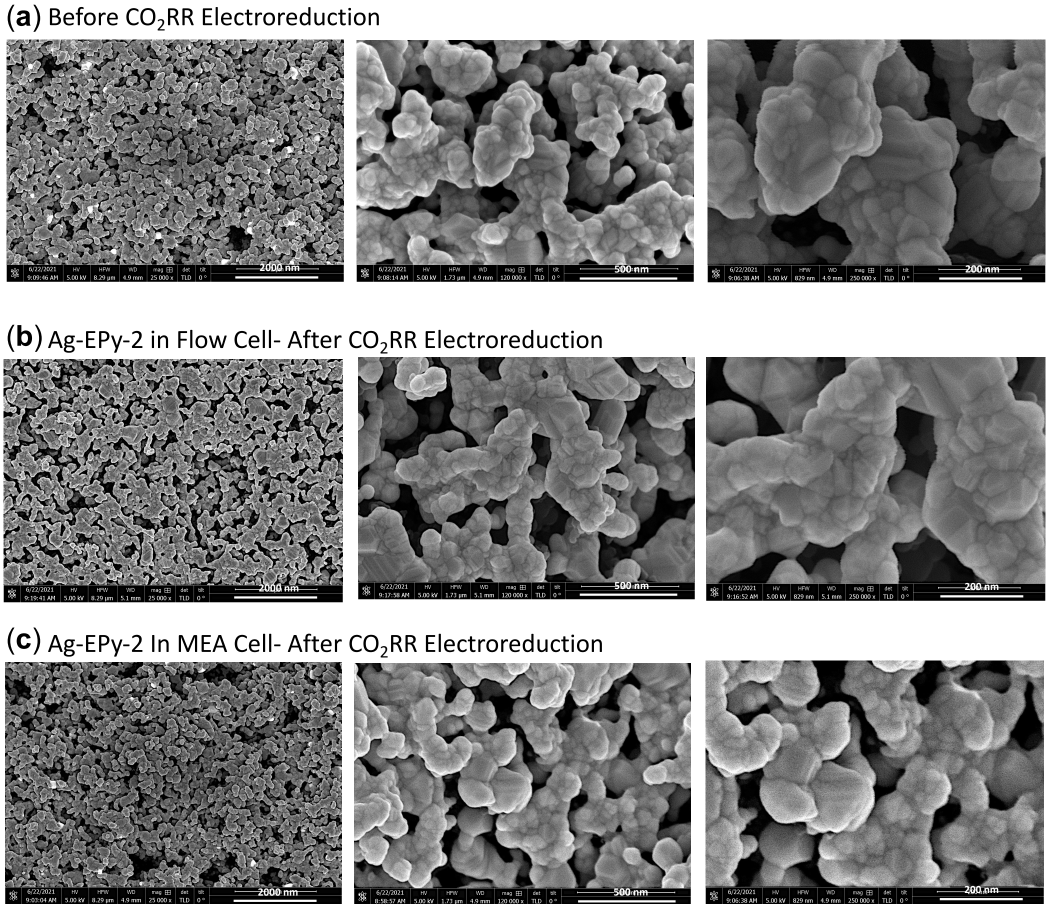

Figure S37. Scanning electron microscopy (SEM) of Ag-Epy-2 (a) before electrochemical CO_2RR; (b) after electrochemical CO_2RR in a gas phase flow cell; (c) after electrochemical CO_2RR in a liquid phase MEA.

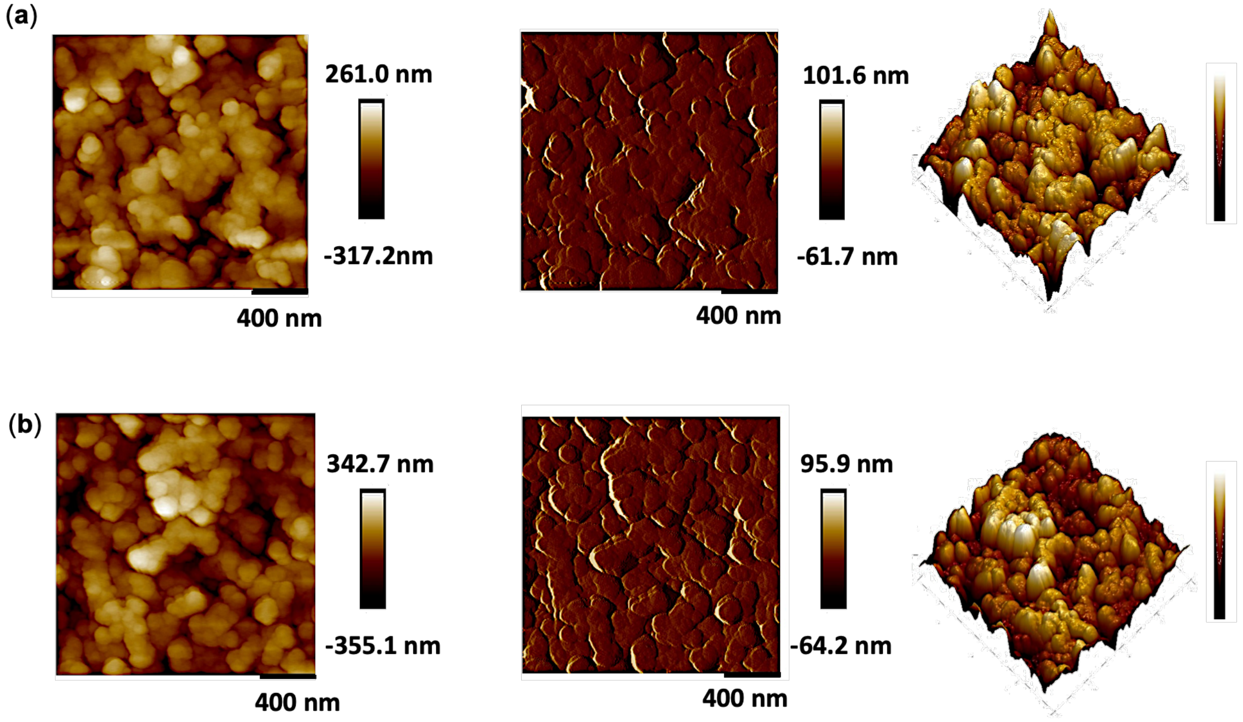

Figure S38. Atomic Force Microscopy (AFM) image of the surface of 10 nm of Ag-EPy-2 film (a) before; and (b) after CO_2RR electroreduction in a flow cell.

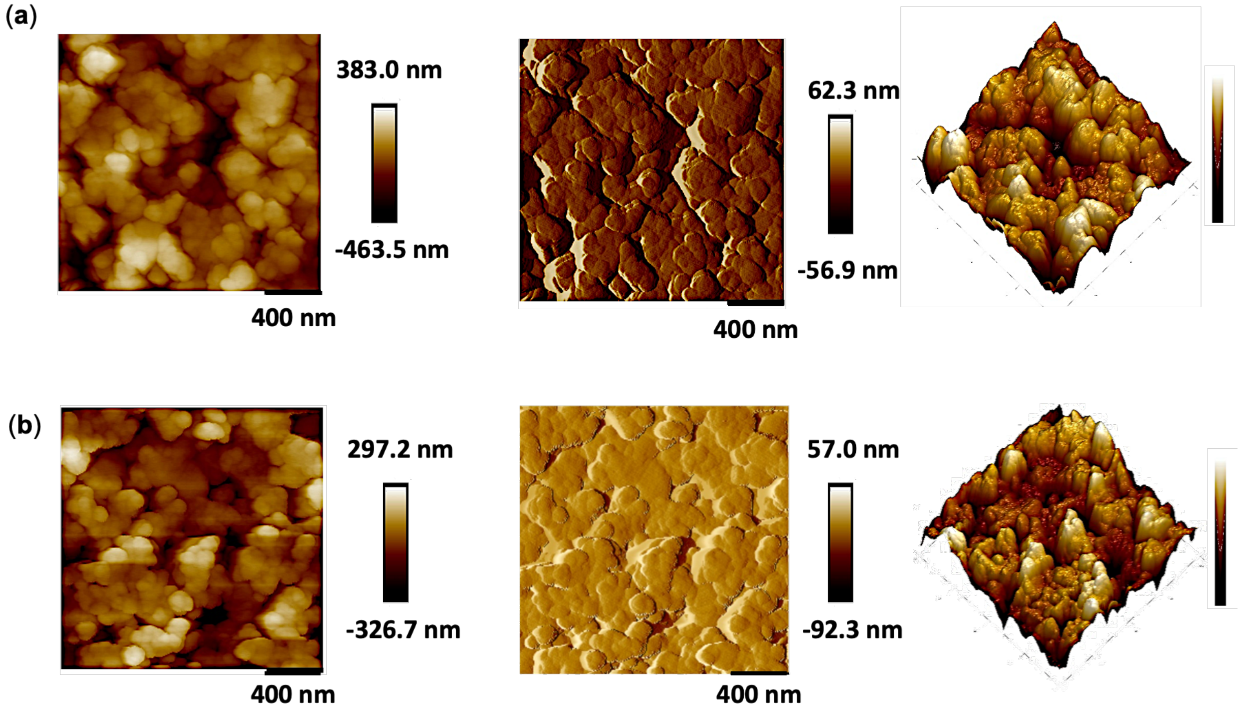

Figure S39. Atomic Force Microscopy (AFM) image of the surface of 10 nm of Ag-EPy-2 film (a) before; and (b) after CO_2RR electroreduction in a MEA cell.

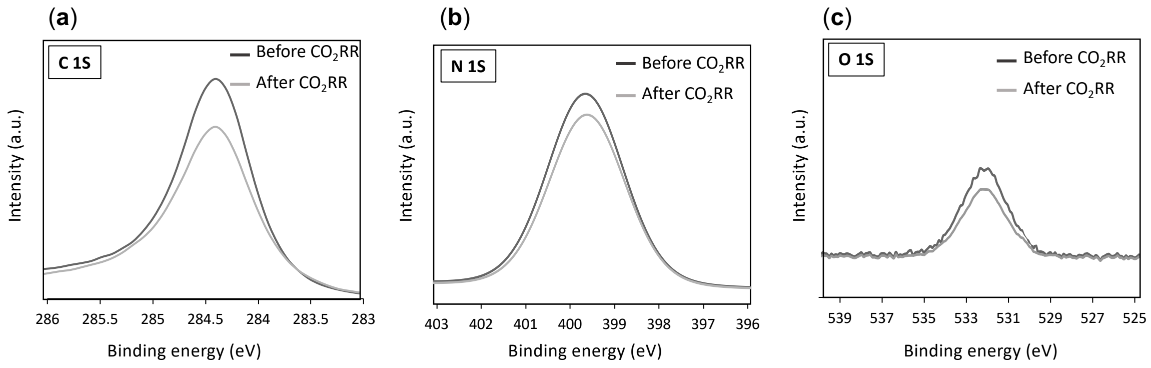

Figure S40. X-ray photoelectron spectroscopy characterization of Ag-EPy-2 before and after electroreduction of CO_2.

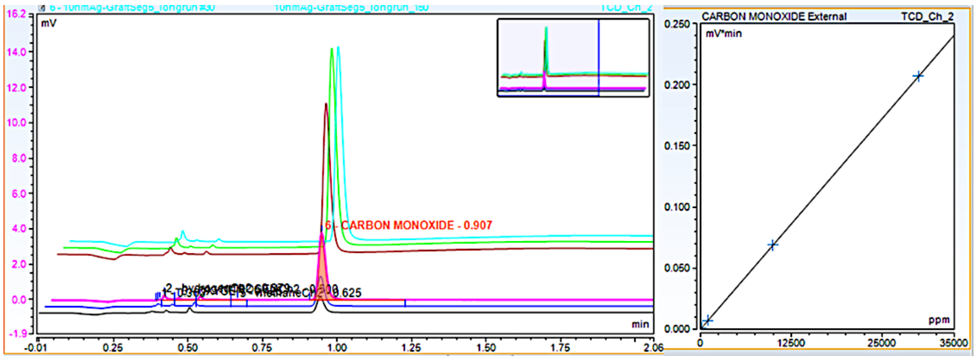

Figure S41. Gas Chromatography (GC) chromatogram example of CO product obtained during CO_2 electroreduction.

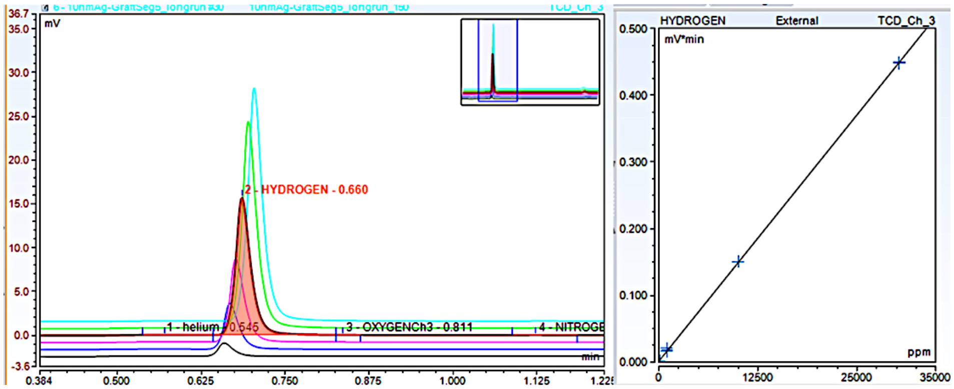

Figure S42. Gas Chromatography (GC) chromatogram of H_2 product obtained during CO_2 electroreduction.

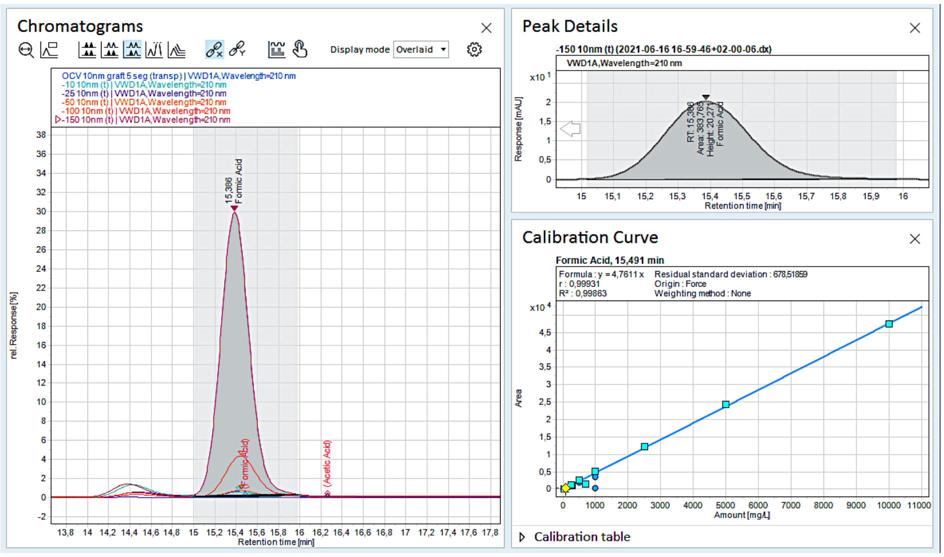

Figure S43. High Performance Liquid Chromatography (HPLC) chromatogram example of formic acid obtained during CO_2 electroreduction.

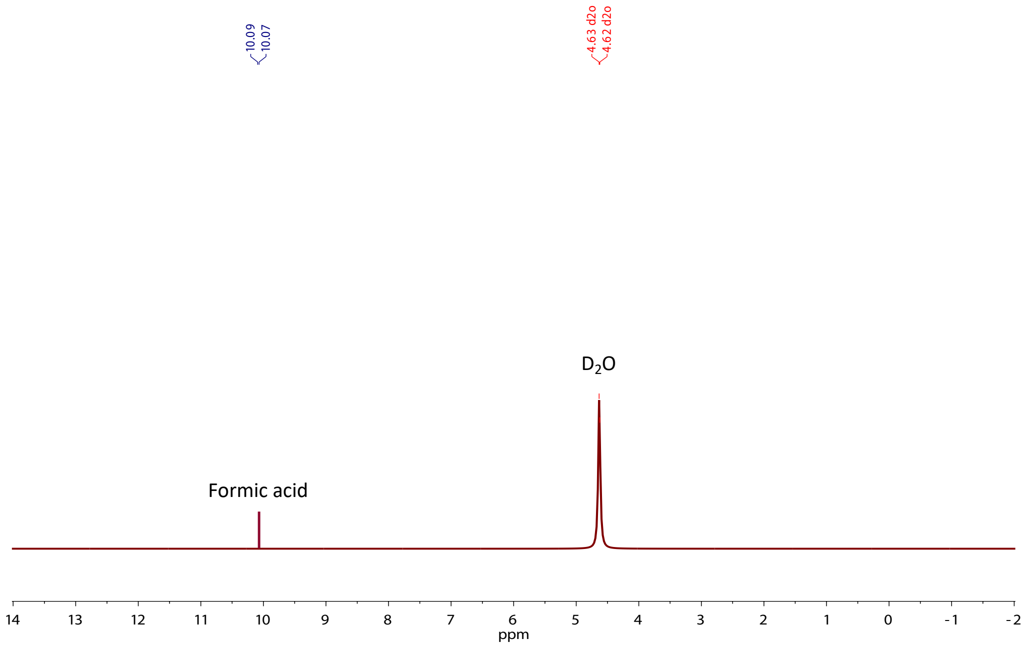

Figure S44. ^{1}H NMR spectrum example of formic acid obtained during CO_2 electroreduction.

Table S5. Product analysis of the homogeneous Py-x, and heterogeneous EPy-x catalysts in H-cell. The reported data are the average values of three separate measurements taken from four individual reaction runs at various potentials.

Compound	Electrolyte	V *vs* RHE	j (mA/cm^2)	FE% (CO)	FE% (Formate)	FE% (H_2)	Ref.
GCE	$KHCO_3$ (0.1 M)	-0.8	~ 0.03	-	-	-	Current work
Homogeneous Py-1	$KHCO_3$ (0.1 M)	-0.5	0.09	-	-	100	Current work
	$KHCO_3$ (0.1 M)	-0.6	0.22	-	-	100	Current work
	$KHCO_3$ (0.1 M)	-0.7	0.39	-	-	97±3.4	Current work
	$KHCO_3$ (0.1 M)	-0.8	0.44	-	-	100	Current work
	$KHCO_3$ (0.1 M)	-0.9	0.61	-	1±1.2	96±2.5	Current work
	$KHCO_3$ (0.1 M)	-1.0	0.65	-	1.2±2	98±1	Current work
Homogeneous Py-2	$KHCO_3$ (0.1 M)	-0.5	0.07	-	-	100	Current work
	$KHCO_3$ (0.1 M)	-0.6	0.22	-	-	100	Current work
	$KHCO_3$ (0.1 M)	-0.7	0.38	-	-	100	Current work
	$KHCO_3$ (0.1 M)	-0.8	0.43	-	-	100	Current work
	$KHCO_3$ (0.1 M)	-0.9	0.61	-	1.3±2.4	97±1.8	Current work
	$KHCO_3$ (0.1 M)	-1.0	0.68	-	1.4±1.7	97±3.4	Current work

Compound	Electrolyte	V *vs* RHE	j (mA/cm^2)	FE% (CO)	FE% (Formate)	FE% (H_2)	Ref.
Homogeneous Py-3	$KHCO_3$ (0.1 M)	-0.5	0.28	-	-	100	Current work
	$KHCO_3$ (0.1 M)	-0.6	0.86	-	-	100	Current work
	$KHCO_3$ (0.1 M)	-0.7	1.72	-	-	100	Current work
	$KHCO_3$ (0.1 M)	-0.8	1.97	-	-	97±0.8	Current work
	$KHCO_3$ (0.1 M)	-0.9	2.4	-	2.2±1.2	96±1.2	Current work
	$KHCO_3$ (0.1 M)	-1.0	3.3	-	2.9±2.4	95±1.7	Current work
Ag	$KHCO_3$ (0.1 M)	-0.5	0.01	-	-	100	Current work
	$KHCO_3$ (0.1 M)	-0.6	0.63	-	-	100	Current work
	$KHCO_3$ (0.1 M)	-0.7	0.9	-	-	100	Current work
	$KHCO_3$ (0.1 M)	-0.8	1.1	10±3.1	-	89±2.8	Current work
	$KHCO_3$ (0.1 M)	-0.9	2.8	36±2.2	-	63±0.9	Current work
	$KHCO_3$ (0.1 M)	-1.0	3.4	14±1	-	85±1.9	Current work
Homogeneous Ag-Py-1	$KHCO_3$ (0.1 M)	-0.5	0.3	-	-	100	Current work
	$KHCO_3$ (0.1 M)	-0.6	0.6	-	-	100	Current work
	$KHCO_3$ (0.1 M)	-0.7	1.37	11±1.9	-	86±1.3	Current work
	$KHCO_3$ (0.1 M)	-0.8	1.76	38±2.1	-	60±1.5	Current work

Compound	Electrolyte	V *vs* RHE	j (mA/cm^2)	FE% (CO)	FE% (Formate)	FE% (H_2)	Ref.
Homogeneous Ag-Py-1	$KHCO_3$ (0.1 M)	-0.9	3.3	-	-	94±2.4	Current work
	$KHCO_3$ (0.1 M)	-1.0	3.5	-	-	100	Current work
Homogeneous Ag-Py-2	$KHCO_3$ (0.1 M)	-0.5	0.2	-	-	100	Current work
	$KHCO_3$ (0.1 M)	-0.6	0.4	-	-	100	Current work
	$KHCO_3$ (0.1 M)	-0.7	1.4	15±2.8	-	84±1.7	Current work
	$KHCO_3$ (0.1 M)	-0.8	1.8	39±1.2	-	58±1.3	Current work
	$KHCO_3$ (0.1 M)	-0.9	3.8	21±3.0	-	76±1.4	Current work
	$KHCO_3$ (0.1 M)	-1.0	4.9	-	-	100	Current work
Homogeneous Ag-Py-3	$KHCO_3$ (0.1 M)	-0.5	0.15	-	-	100	Current work
	$KHCO_3$ (0.1 M)	-0.6	0.63	-	-	98±0.9	Current work
	$KHCO_3$ (0.1 M)	-0.7	1	24±1.1	-	75±1.5	Current work
	$KHCO_3$ (0.1 M)	-0.8	1.7	41±1.9	-	56±3.4	Current work
	$KHCO_3$ (0.1 M)	-0.9	3.9	29±1.3	-	66±1.1	Current work
	$KHCO_3$ (0.1 M)	-1.0	5.6	-	-	100	Current work
Heterogeneous EPy-1	$KHCO_3$ (0.1 M)	-0.5	0.01	-	-	100	Current work
	$KHCO_3$ (0.1 M)	-0.6	0.01	-	-	100	Current work

Compound	Electrolyte	V *vs* RHE	j (mA/cm^2)	FE% (CO)	FE% (Formate)	FE% (H_2)	Ref.
Heterogeneous EPy-1	$KHCO_3$ (0.1 M)	-0.7	0.02	-	-	100	Current work
	$KHCO_3$ (0.1 M)	-0.8	0.16	-	3.2±1.1	96±1.4	Current work
	$KHCO_3$ (0.1 M)	-0.9	0.28	-	-	100	Current work
	$KHCO_3$ (0.1 M)	-1.0	0.51	-	-	100	Current work
Heterogeneous EPy-2	$KHCO_3$ (0.1 M)	-0.5	0.08	-	-	100	Current work
	$KHCO_3$ (0.1 M)	-0.6	0.1	-	-	100	Current work
	$KHCO_3$ (0.1 M)	-0.7	0.23	-	-	100	Current work
	$KHCO_3$ (0.1 M)	-0.8	0.39	-	3.4±2.4	95±1.6	Current work
	$KHCO_3$ (0.1 M)	-0.9	1.1	-	1.2±2.9	98±2.2	Current work
	$KHCO_3$ (0.1 M)	-1.0	1.7	-	-	100	Current work
Heterogeneous EPy-3	$KHCO_3$ (0.1 M)	-0.5	0.01	-	-	100	Current work
	$KHCO_3$ (0.1 M)	-0.6	0.015	-	-	100	Current work
	$KHCO_3$ (0.1 M)	-0.7	0.03	-	-	100	Current work
	$KHCO_3$ (0.1 M)	-0.8	0.15	-	2.8±3.7	97±1.2	Current work
	$KHCO_3$ (0.1 M)	-0.9	0.16	-	-	100	Current work
	$KHCO_3$ (0.1 M)	-1.0	0.52	-	-	100	Current work

Compound	Electrolyte	V *vs* RHE	j (mA/cm^2)	FE% (CO)	FE% (Formate)	FE% (H_2)	Ref.
Heterogeneous Ag-EPy-1	$KHCO_3$ (0.1 M)	-0.5	0.12	-	-	100	Current work
	$KHCO_3$ (0.1 M)	-0.6	0.48	-	-	94±3.3	Current work
	$KHCO_3$ (0.1 M)	-0.7	2.26	69±1.4	-	28±1.2	Current work
	$KHCO_3$ (0.1 M)	-0.8	2.59	54±3.2	-	43±0.8	Current work
	$KHCO_3$ (0.1 M)	-0.9	3.9	36±0.9	-	60±1.2	Current work
	$KHCO_3$ (0.1 M)	-1.0	4.21	19±1.7	-	79±1.7	Current work
Heterogeneous Ag-EPy-2	$KHCO_3$ (0.1 M)	-0.5	0.59	-	-	100	Current work
	$KHCO_3$ (0.1 M)	-0.6	1.22	19±1.1	-	78±2.1	Current work
	$KHCO_3$ (0.1 M)	-0.7	3.1	74±0.9	-	23±0.7	Current work
	$KHCO_3$ (0.1 M)	-0.8	4.1	58±2.4	-	38±1.4	Current work
	$KHCO_3$ (0.1 M)	-0.9	5.8	47±1.3	-	50±1.2	Current work
	$KHCO_3$ (0.1 M)	-1.0	5.6	21±1.7	-	77±1.5	Current work
Heterogeneous Ag-EPy-3	$KHCO_3$ (0.1 M)	-0.5	0.27	-	-	100	Current work
	$KHCO_3$ (0.1 M)	-0.6	0.36	17±1.2	-	82±0.7	Current work
	$KHCO_3$ (0.1 M)	-0.7	1.2	40±0.9	-	54±2	Current work
	$KHCO_3$ (0.1 M)	-0.8	1.9	38±1.2	-	57±1.1	Current work

Compound	Electrolyte	V *vs* RHE	j (mA/cm^2)	FE% (CO)	FE% (Formate)	FE% (H_2)	Ref.
Heterogeneous Ag-EPy-3	$KHCO_3$ (0.1 M)	-0.9	2.6	34±1.7	-	63±1.6	Current work
	$KHCO_3$ (0.1 M)	-1.0	3.0	17±1.1	-	81±1.4	Current work
Ag electrode	$CsHCO_3$ (0.1 M)	-1.0	-5.8	80	-	-	6
Ag	EMIN-BF_4	N/A	-0.61	96	-	4	7
Ag	$KHCO_3$ (0.1 M)	-0.7	-0.4	45	-	18	8
Ag foil	$KHCO_3$ (0.1 M)	-0.8	-0.01	2.2	-	75	8
Ag Nano-coarals	$KHCO_3$ (0.1 M)	-0.7	-6.6	95	-	4	8
Nanoporous Ag	$KHCO_3$ (0.5 M)	-0.8	-0.19	92	-	7	9
Ag Compact grains	$KHCO_3$ (0.1 M)	-1.1	-5.7	89	-	-	10
Ag Plate	$KHCO_3$ (0.1 M)	-1.12	-22.9	79	-	-	11
Ag foam	$KHCO_3$ (0.1 M)	-1.12	-27.43	83	-	-	11
Ag Truncated hexagonal bipyramidal	$KHCO_3$ (0.1 M)	-0.93	-4.92	89	-	-	12
L25-Ag nanocubes	$KHCO_3$ (0.1 M)	-0.85	-1.7	99	-	-	13
D-25 Ag NWs (diameter less than 25 nm)	$KHCO_3$ (0.1 M)	-0.96	-3.2	99	-	-	14
Ag NWs (35 nm)	$KHCO_3$ (0.5 M)	-0.9	-7	80	-	-	15
Ag NWs (200 nm)	$KHCO_3$ (0.5 M)	-0.7	-12.2	84	-	-	16

Compound	Electrolyte	V *vs* RHE	j (mA/cm^2)	FE% (CO)	FE% (Formate)	FE% (H_2)	Ref.
6 µm thick highly porous Ag	$KHCO_3$ (0.5 M)	-0.5	-10.5	82	-	-	[17]
Sponge-like porous Ag	$KHCO_3$ (0.1 M)	-0.9	-7	93	-	-	[18]
Ag nanosheets	$KHCO_3$ (0.5 M)	-0.6	-1.6	90	-	-	[19]
AgCl-derived Ag	NaCl (3.5%)	-1.1	-7.5	90	-	-	[20]
Ag3PO4-derived Ag	$KHCO_3$ (0.5 M)	-0.7	-2.93	97.3	-	-	[21]
Iodide-derived Ag	$KHCO_3$ (0.5 M)	-0.7	-16.7	94.5	-	-	[22]
Ag2P nano crystals	$KHCO_3$ (0.5 M)	-0.8	-7.5	82	-	-	[23]
cysteamine Ag	$KHCO_3$ (0.5 M)	-0.75	-3.8	84.4	-	-	[24]
Benzenethiolate -modified	$KHCO_3$ (0.1 M)	-1.03	-502/g	96	-	-	[25]
Polycrystalline Ag	$KHCO_3$ (0.5 M)	-0.75	-1.0	70.5	-	28	[24]
polycrystalline Ag electrode	$NaNO_3$ (0.1 M)	-0.6	-3.7	92.8	-	-	[26]
Thiol Modified Ag/C	$KHCO_3$ (0.5 M)	-1.0	-0.15	65.5	-	35	[27]
Amine Derived-Pb	$KHCO_3$ (1 M)	-1.09	-9.5	94	-	6	[28]

Table S6. Product analysis of heterogeneous EPy-2 and Ag-EPy-2 catalysts in a MEA Cell.

Compound	Electrolyte	V (Cell voltage)	j (mA/cm^2)	FE% (CO)	FE% (Formate)	FE% (H_2)	Ref.
Ag	KOH (1 M)	2.4	25	83±1	6.6±0.9	4.4±1	Current work
	KOH (1 M)	2.5	50	79±1	6.7±0.8	4.9±1	Current work
	KOH (1 M)	2.7	100	74±1.3	10±0.7	5.1±0.6	Current work
	KOH (1 M)	2.8	150	71±0.5	22.5±0.9	6.1±0.7	Current work
	KOH (1 M)	2.8	200	68±0.8	11.9±0.5	22.8±0.9	Current work
Ag-EPy-2	KOH (1 M)	2.3	25	87±0.2	4.67	3.5±1.6	Current work
	KOH (1 M)	2.4	50	84±0.8	5.19	3.77±1.7	Current work
	KOH (1 M)	2.6	100	77±0.9	8.6±1	4.12±0.7	Current work
	KOH (1 M)	2.7	150	71±0.8	9.81±0.5	6.19±0.8	Current work
	KOH (1 M)	2.8	200	61±1	10.42±0.7	19±0.9	Current work
EPy-2 (No Silver)	KOH (1 M)	2.4	25	0.93±0.9	2.4±0.8	-	Current work
	KOH (1 M)	2.5	50	1.67±0.8	1.1±0.5	0.9	Current work
	KOH (1 M)	2.6	100	0.64±1	2.3±1	2.1	Current work
	KOH (1 M)	2.8	150	0.64±0.4	2.4±1	2.4	Current work
	KOH (1 M)	2.9	200	-	-	-	-

Table S7. Product analysis of heterogeneous Ag-EPy-2 in a Flow Cell.

Compound	Electrolyte	V *vs* RHE	j (mA/cm^2)	FE% (CO)	FE% (Formate)	FE% (H_2)	Ref.
Ag	$KHCO_3$ (0.1 M)	-1.9	10	75±1.5	0	13±1	Current work
	$KHCO_3$ (0.1 M)	-2.4	25	78±1	`9.1	12±1	Current work
	$KHCO_3$ (0.1 M)	-2.7	50	72±3.1	10.5	18±3	Current work
	$KHCO_3$ (0.1 M)	-3.8	100	46±2.9	13.8	41±3	Current work
	$KHCO_3$ (0.1 M)	-4.2	150	30±2.6	13.6	55±1.4	Current work
Ag-EPy-2	$KHCO_3$ (0.1 M)	-1.7	10	46±2.9	-	40±1	Current work
	$KHCO_3$ (0.1 M)	-2.1	25	44±1	2.5	47±1	Current work
	$KHCO_3$ (0.1 M)	-2.7	50	49±1	2.7	44±3	Current work
	$KHCO_3$ (0.1 M)	-3.7	100	46±1	4.2	38±3.6	Current work
	$KHCO_3$ (0.1 M)	-4.2	150	58±1	3.3	33±1.4	Current work

References:

(1) Lucio, A. J.; Shaw, S. K. Pyridine and Pyridinium Electrochemistry on Polycrystalline Gold Electrodes and Implications for CO_2 Reduction. *J. Phys. Chem. C* **2015**, *119* (22), 12523–12530. https://doi.org/10.1021/acs.jpcc.5b03355.

(2) Peroff, A. G.; Weitz, E.; Van Duyne, R. P. Mechanistic Studies of Pyridinium Electrochemistry: Alternative Chemical Pathways in the Presence of CO_2. *Phys. Chem. Chem. Phys.* **2016**, *18* (3), 1578–1586. https://doi.org/10.1039/C5CP04757A.

(3) Portenkirchner, E.; Enengl, C.; Enengl, S.; Hinterberger, G.; Schlager, S.; Apaydin, D.; Neugebauer, H.; Knör, G.; Sariciftci, N. S. A Comparison of Pyridazine and Pyridine as Electrocatalysts for the Reduction of Carbon Dioxide to Methanol. *ChemElectroChem* **2014**, *1* (9), 1543–1548. https://doi.org/https://doi.org/10.1002/celc.201402132.

(4) Ngamchuea, K.; Eloul, S.; Tschulik, K.; Compton, R. G. Planar Diffusion to Macro Disc Electrodes—What Electrode Size Is Required for the Cottrell and Randles-Sevcik Equations to Apply Quantitatively? *J. Solid State Electrochem.* **2014**, *18* (12), 3251–3257. https://doi.org/10.1007/s10008-014-2664-z.

(5) Mishyn, V.; Aspermair, P.; Leroux, Y.; Happy, H.; Knoll, W.; Boukherroub, R.; Szunerits, S. "Click" Chemistry on Gold Electrodes Modified with Reduced Graphene Oxide by Electrophoretic Deposition. *Surfaces* . **2019**, *2* (1), 193-204. https://doi.org/10.3390/surfaces2010015.

(6) Singh, M. R.; Kwon, Y.; Lum, Y.; Ager, J. W.; Bell, A. T. Hydrolysis of Electrolyte Cations Enhances the Electrochemical Reduction of CO_2 over Ag and Cu. *J. Am. Chem. Soc.* **2016**, *138* (39), 13006–13012. https://doi.org/10.1021/jacs.6b07612.

(7) Rosen, B. a; Salehi-khojin, A.; Thorson, M. R.; Zhu, W.; Whipple, D. T.; Kenis, P. J. a; Masel, R. I. Ionic Liquid – Mediated Selective Conversion of CO_2 to CO at Low Overpotentials. *Science.* **2011**, *334*, 643–644. https://doi.org/10.1126/science.1209786.

(8) Hsieh, Y.-C.; Senanayake, S. D.; Zhang, Y.; Xu, W.; Polyansky, D. E. Effect of Chloride Anions on the Synthesis and Enhanced Catalytic Activity of Silver Nanocoral Electrodes for CO_2 Electroreduction. *ACS Catal.* **2015**, *5* (9), 5349–5356. https://doi.org/10.1021/acscatal.5b01235.

(9) Lu, Q.; Rosen, J.; Zhou, Y.; Hutchings, G. S.; Kimmel, Y. C.; Chen, J. G.; Jiao, F. A Selective and Efficient Electrocatalyst for Carbon Dioxide Reduction. *Nat. Commun.* **2014**, *5* (1), 3242. https://doi.org/10.1038/ncomms4242.

(10) Jianping, Q.; Juntao, T.; Jie, S.; Cuiwei, W.; Mengqian, Q.; Zhiqiao, H.; Jianmeng, C.; Song, S. Preparation of a Silver Electrode with a Three-Dimensional Surface and Its Performance in the Electrochemical Reduction of Carbon Dioxide. *Electrochim. Acta* **2016**, *203*, 99–108.

https://doi.org/https://doi.org/10.1016/j.electacta.2016.03.182.
(11) Yu, Y.; Zhong, N.; Fang, J.; Tang, S.; Ye, X.; He, Z.; Song, S. Comparative Study between Pristine Ag and Ag Foam for Electrochemical Synthesis of Syngas with Carbon Dioxide and Water. *Catalysts*. 2019, p 57. https://doi.org/10.3390/catal9010057.
(12) He, Z.; Liu, T.; Tang, J.; Zhou, C.; Wen, L.; Chen, J.; Song, S. Highly Active, Selective and Stable Electroreduction of Carbon Dioxide to Carbon Monoxide on a Silver Catalyst with Truncated Hexagonal Bipyramidal Shape. *Electrochim. Acta* **2016**, *222*, 1234–1242. https://doi.org/https://doi.org/10.1016/j.electacta.2016.11.097.
(13) Liu, S.; Sun, C.; Xiao, J.; Luo, J.-L. Unraveling Structure Sensitivity in CO_2 Electroreduction to Near-Unity CO on Silver Nanocubes. *ACS Catal.* **2020**, *10* (5), 3158–3163. https://doi.org/10.1021/acscatal.9b03883.
(14) Liu, S.; Wang, X.-Z.; Tao, H.; Li, T.; Liu, Q.; Xu, Z.; Fu, X.-Z.; Luo, J.-L. Ultrathin 5-Fold Twinned Sub-25nm Silver Nanowires Enable Highly Selective Electroreduction of CO_2 to CO. *Nano Energy* **2018**, *45*, 456–462. https://doi.org/https://doi.org/10.1016/j.nanoen.2018.01.016.
(15) Xi, W.; Ma, R.; Wang, H.; Gao, Z.; Zhang, W.; Zhao, Y. Ultrathin Ag Nanowires Electrode for Electrochemical Syngas Production from Carbon Dioxide. *ACS Sustain. Chem. Eng.* **2018**, *6* (6), 7687–7694. https://doi.org/10.1021/acssuschemeng.8b00527.
(16) Luan, C.; Shao, Y.; Lu, Q.; Gao, S.; Huang, K.; Wu, H.; Yao, K. High-Performance Carbon Dioxide Electrocatalytic Reduction by Easily Fabricated Large-Scale Silver Nanowire Arrays. *ACS Appl. Mater. Interfaces* **2018**, *10* (21), 17950–17956. https://doi.org/10.1021/acsami.8b03461.
(17) Zhang, L.; Wang, Z.; Mehio, N.; Jin, X.; Dai, S. Thickness- and Particle-Size-Dependent Electrochemical Reduction of Carbon Dioxide on Thin-Layer Porous Silver Electrodes. *ChemSusChem* **2016**, *9* (5), 428–432. https://doi.org/https://doi.org/10.1002/cssc.201501637.
(18) Fan, T.; Wu, Q.; Yang, Z.; Song, Y.; Zhang, J.; Huang, P.; Chen, Z.; Dong, Y.; Fang, W.; Yi, X. Electrochemically Driven Formation of Sponge-Like Porous Silver Nanocubes Toward Efficient CO_2 Electroreduction to CO. *ChemSusChem* **2020**, *13* (10), 2677–2683. https://doi.org/https://doi.org/10.1002/cssc.201903558.
(19) Lee, C.-Y.; Zhao, Y.; Wang, C.; Mitchell, D. R. G.; Wallace, G. G. Rapid Formation of Self-Organised Ag Nanosheets with High Efficiency and Selectivity in CO_2 Electroreduction to CO. *Sustain. Energy Fuels* **2017**, *1* (5), 1023–1027. https://doi.org/10.1039/C7SE00069C.
(20) Lee, C.-Y.; Wallace, G. G. CO_2 Electrolysis in Seawater: Calcification Effect and a Hybrid Self-Powered Concept. *J. Mater. Chem. A* **2018**, *6* (46), 23301–23307. https://doi.org/10.1039/C8TA09368G.
(21) Gao, J.; Zhu, C.; Zhu, M.; Fu, Y.; Huang, H.; Liu, Y.; Kang, Z. Highly Selective and Efficient

Electroreduction of Carbon Dioxide to Carbon Monoxide with Phosphate Silver-Derived Coral-like Silver. *ACS Sustain. Chem. Eng.* **2019**, *7* (3), 3536–3543. https://doi.org/10.1021/acssuschemeng.8b05776.

(22) Zhang, Y.; Ji, L.; Qiu, W.; Shi, X.; Asiri, A. M.; Sun, X. Iodide-Derived Nanostructured Silver Promotes Selective and Efficient Carbon Dioxide Conversion into Carbon Monoxide. *Chem. Commun.* **2018**, *54* (21), 2666–2669. https://doi.org/10.1039/C8CC00984H.

(23) Li, H.; Wen, P.; Itanze, D. S.; Hood, Z. D.; Ma, X.; Kim, M.; Adhikari, S.; Lu, C.; Dun, C.; Chi, M.; Qiu, Y.; Geyer, S. M. Colloidal Silver Diphosphide (AgP2) Nanocrystals as Low Overpotential Catalysts for CO_2 Reduction to Tunable Syngas. *Nat. Commun.* **2019**, *10* (1), 5724. https://doi.org/10.1038/s41467-019-13388-8.

(24) Kim, C.; Jeon, H. S.; Eom, T.; Jee, M. S.; Kim, H.; Friend, C. M.; Min, B. K.; Hwang, Y. J. Achieving Selective and Efficient Electrocatalytic Activity for CO_2 Reduction Using Immobilized Silver Nanoparticles. *J. Am. Chem. Soc.* **2015**, *137* (43), 13844–13850. https://doi.org/10.1021/jacs.5b06568.

(25) Abeyweera, S. C.; Yu, J.; Perdew, J. P.; Yan, Q.; Sun, Y. Hierarchically 3D Porous Ag Nanostructures Derived from Silver Benzenethiolate Nanoboxes: Enabling CO_2 Reduction with a Near-Unity Selectivity and Mass-Specific Current Density over 500 A/G. *Nano Lett.* **2020**, *20* (4), 2806–2811. https://doi.org/10.1021/acs.nanolett.0c00518.

(26) Zhou, L. Q.; Ling, C.; Jones, M.; Jia, H. Selective CO_2 Reduction on a Polycrystalline Ag Electrode Enhanced by Anodization Treatment. *Chem. Commun.* **2015**, *51* (100), 17704–17707. https://doi.org/10.1039/C5CC06752A.

(27) Kim, C.; Eom, T.; Jee, M. S.; Jung, H.; Kim, H.; Min, B. K.; Hwang, Y. J. Insight into Electrochemical CO_2 Reduction on Surface-Molecule-Mediated Ag Nanoparticles. *ACS Catal.* **2017**, *7* (1), 779–785. https://doi.org/10.1021/acscatal.6b01862.

(28) Zouaoui, N.; Ossonon, B. D.; Fan, M.; Mayilukila, D.; Garbarino, S.; de Silveira, G.; Botton, G. A.; Guay, D.; Tavares, A. C. Electroreduction of CO_2 to Formate on Amine Modified Pb Electrodes. *J. Mater. Chem. A* **2019**, *7* (18), 11272–11281. https://doi.org/10.1039/C8TA09637F.
